# Supplementary material for: Multi-omics analysis reveals COVID-19 vaccine induced attenuation of inflammatory responses during breakthrough disease
Source: Nat Commun. 2024 Apr 22;15:3402. doi: 10.1038/s41467-024-47463-6 (PMC11035709; doi:10.1038/s41467-024-47463-6)
Supplement: Supplementary file 1 — Supplementary Information [file 41467_2024_47463_MOESM1_ESM.pdf]

Supplementary

Supplementary Methods

Assignment of multi-mapping reads in the small RNA sequencing data.

Multi-mapping features are common in small RNA sequencing due to the shortness of the sequences, embedding of miRNA within the introns of mRNAs and non-coding RNAs and the frequent duplication of non-coding RNAs in the genome. To deal with multi-mapping reads, a gene_union approach was used. In this approach, a multi-mapping sequence is assigned to a newly created feature whose name is a concatenation of all the genes it maps to. Sequences which map to the same features will be collapsed to the same newly created feature. The advantage of this is that all potential loci are represented in the gene expression table, redundancy is minimised (because reads only contribute to one feature in the gene expression table), and feature names retain information on mapping certainty, so if feature A_B is differentially expressed, it is clear that this sequence could have arisen from either or both genes.

Reads were assigned to the non-coding RNA. The bowtie indexes for the non-coding RNAome were built by merging fasta files from the following databases: miRbase (miRNAs), SnOPY (snoRNAs), trRNA_db (tRNAs), RefSeq (snRNAs, yRNAs, vault RNAs, lncRNAs)[1-3]. piRNA annotations are not included because there are no high-quality piRNA repositories – most repositories contain sequences whose mapping is ambiguous (i.e. many map to other non-coding RNAs). mRNA transcript sequences were downloaded from GENCODE.

miRNA enrichment analyses

Integrating lists of miRNAs and mRNAs

miRNA set enrichment analysis:

miRNA set enrichment analysis asks whether a single mRNA is targeted by miRNAs that are generally up or down-regulated, i.e. miRNAs that appear towards the top or bottom of a ranked list of miRNA (in this study, a differential miRNA expression list ranked by their t-statistic from most upregulated to most downregulated). Negative enrichment means that miRNAs towards the bottom of that miRNA-ranked list target the mRNA. Positive enrichment means miRNAs towards the top of that miRNA-ranked list target the mRNA.

In this study, positive enrichment predicts that the expression of the mRNA (or its protein) would decrease due to more targeting by miRNAs. Conversely, negative enrichment would suggest that the expression of the mRNA (or its protein) would increase due to less targeting by miRNAs. The MIEAA 2.0 web server was used to implement this strategy[4].

miRNA target enrichment analysis

miRNA target enrichment analysis asks whether the mRNA targets of a miRNA are generally up or down-regulated, i.e. whether they appear towards the top or bottom of a ranked list of miRNA (in this study, the differential mRNA expression list was ranked by their t-statistic from most upregulated to most downregulated).

Positive enrichment means a miRNA targets mRNAs towards the top of that ranked list. Conversely, negative enrichment means a miRNA targets mRNAs towards the bottom of that ranked list.
If there is a relationship between mRNAs and miRNAs in a system, upregulated miRNAs should have targets which are negatively enriched because those targets are subject to increased repression. Conversely, downregulated miRNAs should have targets which are positively enriched because those targets are released from repression. This strategy is implemented in R using miRNA target interactions downloaded from miRnet, and the fsgea package, which implements a GSEA-style enrichment analysis[5,6].

miRNA pathway analysis

MIEAA2 GSEA style analysis

This approach asks whether all the miRNAs that target all the mRNAs in a pathway are generally up or downregulated. Positive enrichment means that the pathway contains mRNAs targeted by miRNAs that are mainly towards the top of the miRNA differential expression list (ranked by t-statistic from most upregulated to most downregulated). Negative enrichment means that the pathway contains mRNAs targeted by miRNAs towards the bottom of that ranked list. Positive enrichment predicts that the expression of mRNAs/proteins in that pathway would decrease due to more targeting by miRNAs. Conversely, negative enrichment predicts that the expression of mRNAs/proteins in that pathway increases due to less targeting by miRNAs. MiRNA pathway analysis was done using the MIEAA2 web server[4].

A different way of performing miRNA pathway enrichment analysis is to convert a miRNA(s) to the genes they target and perform a pathway over-representation analysis. Although this approach is common in the literature, it generates less useful terms, and cell-cycle and cancer terms are often over-represented regardless of the list of inputted miRNAs[7]. In addition, a GSEA-style analysis cannot be done. For this reason, MIEAA2 was used for miRNA pathway-enriched analyses.

Supplementary Results

Supplementary Figures

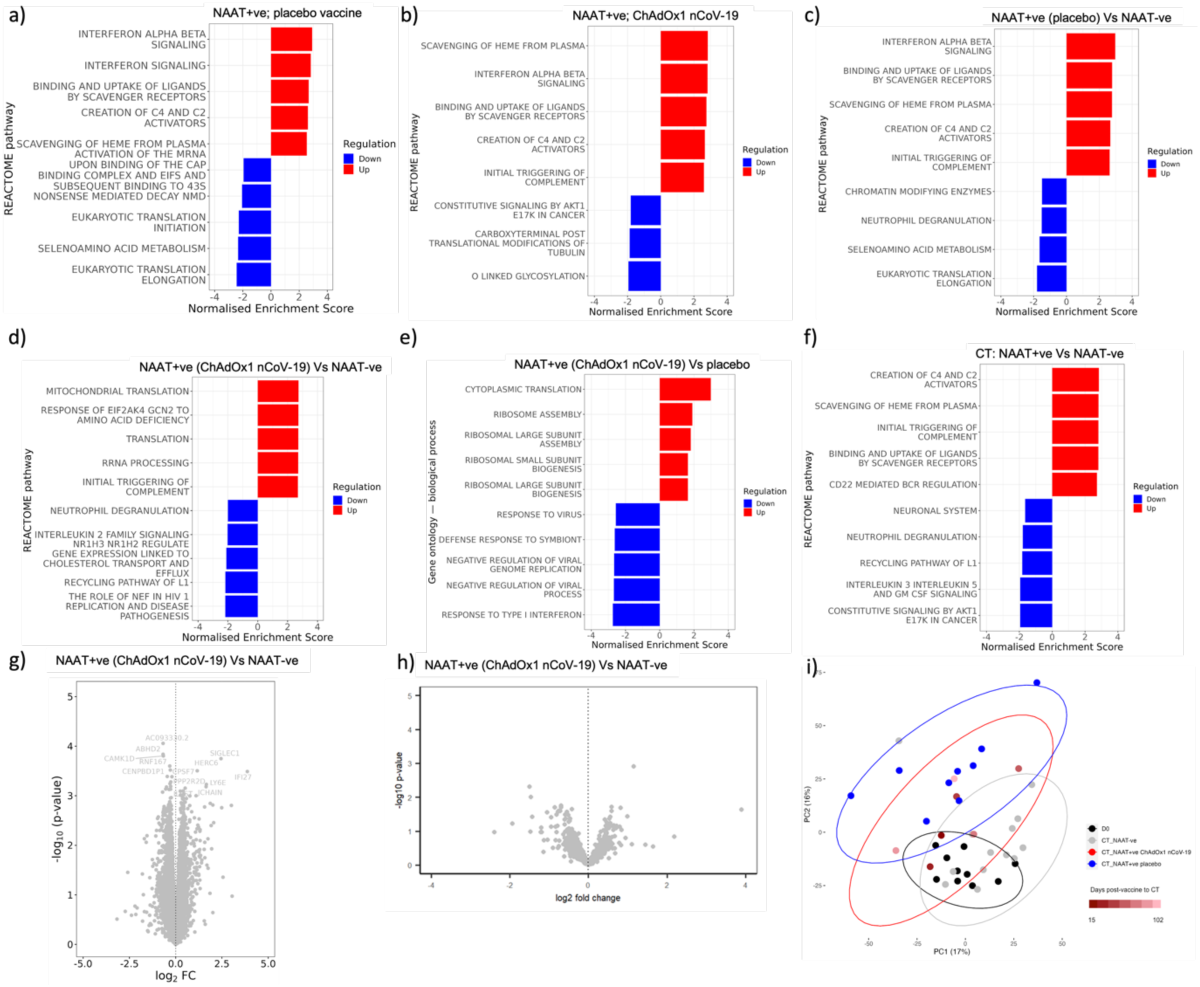

Supplementary figure 1: Comparing COVID-19 with health and COVID-like illness at gene and pathway levels. a) Gene set enrichment analysis of symptomatic (GSEA) — REACTOME— NAAT+ve individuals from the placebo vaccine arm (n=9). b) GSEA — REACTOME NAAT+ve individuals from the ChAdOx1 nCoV-19 vaccine arm (n=7). c) GSEA — REACTOME — NAAT+ve (placebo vaccine, n=9) compared with symptomatic NAAT-ve individuals (n=13). d) GSEA — REACTOME — NAAT+ve (ChAdOx1 nCoV-19 vaccine, n=9) compared with symptomatic NAAT-ve individuals (n=13). e) Gene set enrichment analysis (GSEA) gene ontology biological process ChAdOx1 nCoV-19 versus placebo vaccine NAAT+ve individuals. f) GSEA —REACTOME— NAAT+ve (both vaccine groups, n=16) vs NAAT-ve individuals (n=13). g) Volcano plot comparing the next-gen RNA-seq blood transcriptome of NAAT+ve (ChAdOx1 nCoV-19 vaccine, n=7) and NAAT-ve individuals (n=13) at CT. Differential expression analysis was performed using a two-sided moderate t-test. h) Volcano plot comparing the small RNA-seq blood transcriptome of NAAT+ve (ChAdOx1 nCoV-19 vaccine n=7) and symptomatic NAAT-ve individuals at CT (n=13). Differential expression analysis was performed using a two-sided moderate t-test. i) Principal component analysis of blood RNA-seq transcriptome (next-gen RNA-seq) of study participants during symptomatic episodes consistent with COVID-19, with 95% confidence intervals ellipses, stage 1 data. D0 n=10, CT NAAT-ve n=13, CT NAAT+ve ChAdOx1 nCoV-19 n=7, CT NAAT+ve placebo n=9. Time from the last ChAdOx1 nCoV-19 vaccination until CT in NAAT+ve group is shown by the colour gradient.

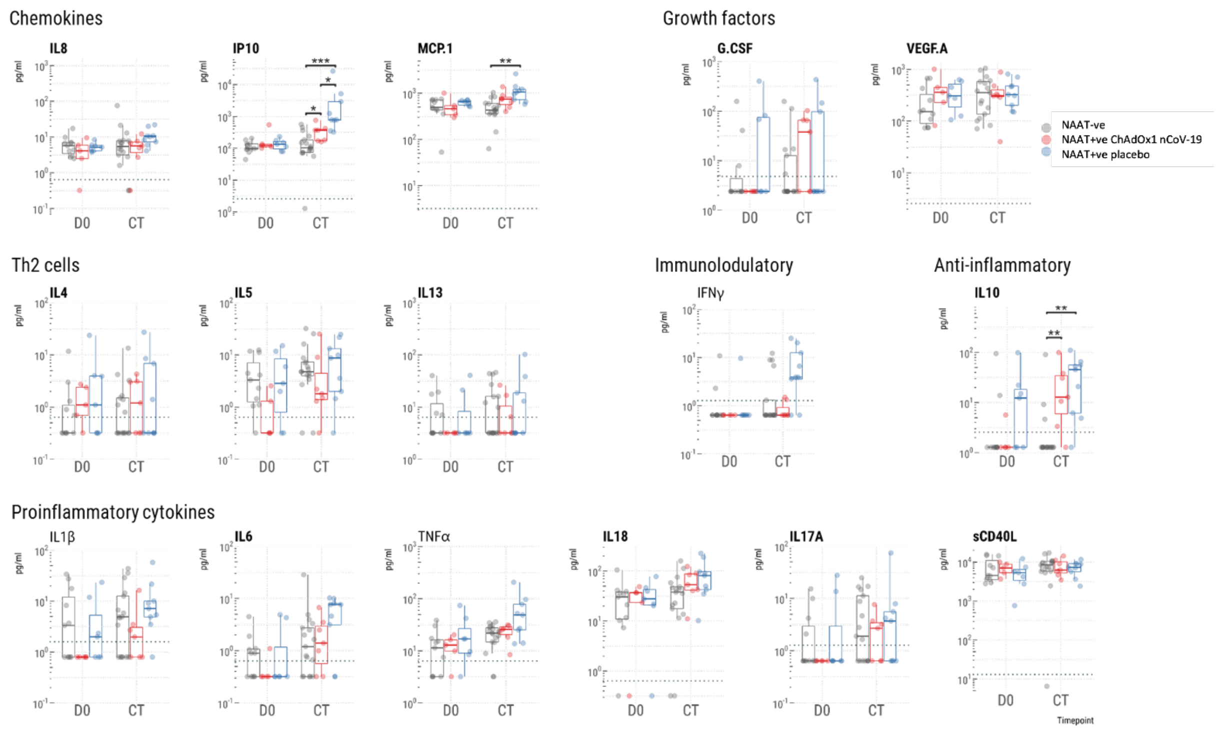

Supplementary figure 2: Serum concentrations of 16 detectable cytokines (Chemokines IL8, IP10 and MCP1, growth factors G.CSF and VEGF.A, type 2 cytokines IL4, IL5 and IL13, immunomodulatory IFNγ, anti-inflammatory cytokine IL10 and pro-inflammatory cytokines IL1β, IL6, TNFα, IL18, IL17A and sCD40L) measured by Luminex on Stage 1 before vaccinations (D0) and around symptom onset (CT) in NAAT-ve (grey, D0 n=11, CT n=17), NAAT+ve receiving ChAdOx1 nCoV-19 vaccine (red, D0 n=5, CT n=7) and NAAT+ve who received the placebo vaccine (blue, D0 n=7, CT n=9). Each dot represents a volunteer, displayed with medians and IQRs. The dotted line indicates the limit of detection, and values below the limit of detection were assigned a value of half the limit of detection. Statistical comparisons were applied among the 3 groups at each time point by two-sided unpaired Wilcoxon test with FDR test for adjusting, shown as *FDR < 0.05, **FDR < 0.01, ***FDR < 0.001.

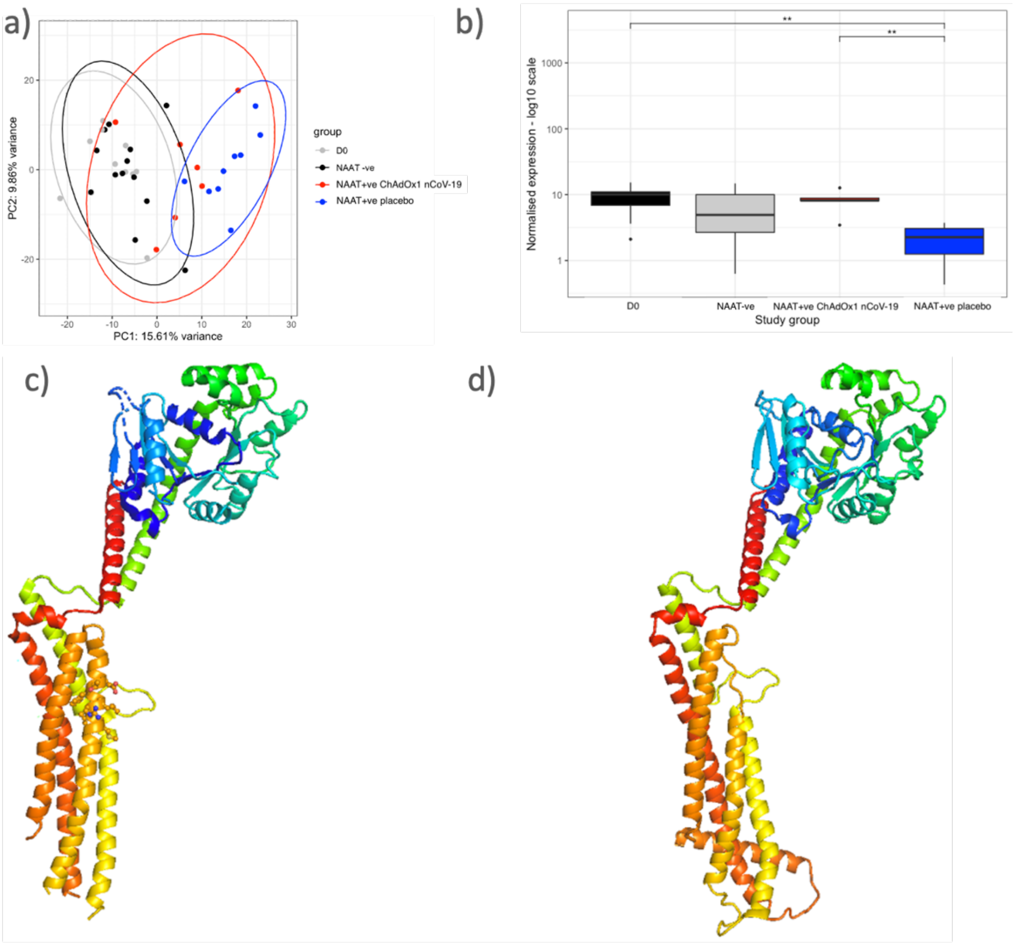

Supplementary figure 3: Comparing COVID-19 with health and non-covid-like illness in the long-read sequencing data. a) Principal component analysis of long-read 3rd gen ONT RNA-seq blood transcriptome of study participants during symptomatic episode consistent with COVID-19, with 95% confidence intervals ellipses. D0 n=10, CT NAAT-ve n=13, CT NAAT+ve ChAdOx1 nCoV-19 n=7, CT NAAT+ve placebo n=9. b) CHCHD5 ENST00000409719 transcript (CHCHD5-202 isoform) expression across the study groups. Statistics: unpaired Wilcoxon test; only significant Bonferroni p-adjusted values are shown (D0 vs NAAT+ve MenACWY p.adj=0.003774, NAAT+ve ChAdOx1 vs NAAT+ve MenACWY p.adj=0.006). c) and d) MX1 protein structure: c) Crystal structure of canonical, wild type 662 aa MX1 protein[8]. Side chains of Met479-VRLAFT-Asp486 are shown as sticks and spheres; d) AlphaFold-derived model of the predicted structure of the truncated 654 aa protein isoform[9].

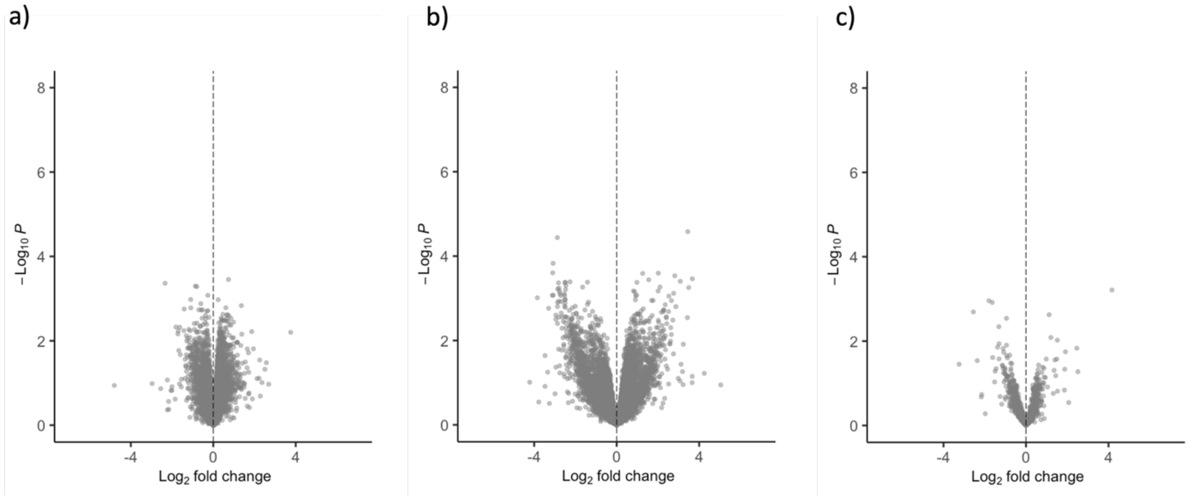

Supplementary figure 4: Confirmation that vaccination makes no difference in COVID-like illness. a) Volcano plot comparing the next-gen RNA-seq blood transcriptome of NAAT-ve ChAdOx1 nCoV-19 vaccinees (n=5) and NAAT-ve Placebo individuals (n=8) at CT. b) Volcano plot comparing the 3rd gen RNA-seq blood transcriptome of NAAT-ve ChAdOx1 nCoV-19 vaccinees (n=5) and NAAT-ve Placebo individuals (n=8) at CT. c) Volcano plot comparing the small RNA-seq blood transcriptome of NAAT-ve ChAdOx1 nCoV-19 vaccinees (n=5) and NAAT-ve Placebo individuals (n=8) at CT. Differential expression analysis was performed using a two-sided moderate t-test. Source data provided in source data file.

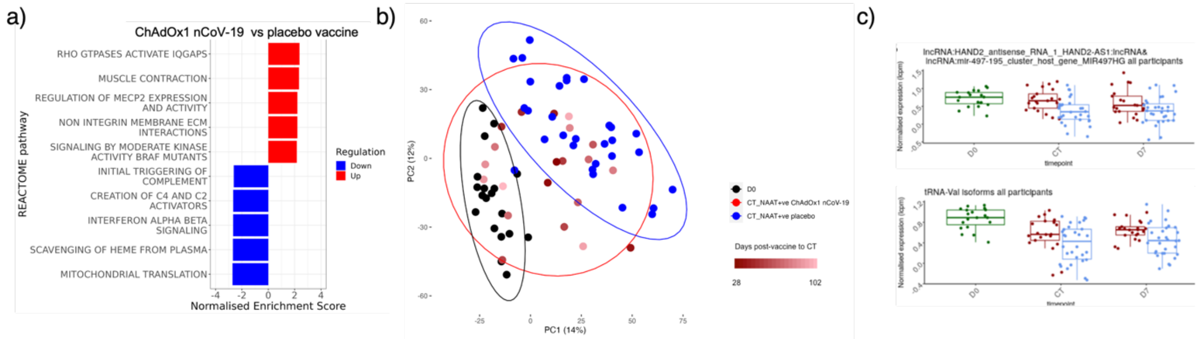

Supplementary figure 5: Differences between ChAdOx1 nCoV-19 and placebo vaccinees at COVID-19 in the Stage 2 data. a) Gene set enrichment analysis (GSEA) REACTOME pathway ChAdOx1 nCoV-19 versus placebo vaccine NAAT+ve individuals. CT NAAT+ve ChAdOx1 nCoV-19 n=21, CT NAAT+ve placebo n=30. b) Principal component analysis of blood RNA-seq transcriptome (next-gen RNA-seq) of study participants during symptomatic episodes consistent with COVID-19, with 95% confidence intervals of data points shown in ellipses, stage 2 data. D0 n=19, CT NAAT+ve ChAdOx1 nCoV-19 n=21, CT NAAT+ve placebo n=30. Time from the last ChAdOx1 nCoV-19 vaccination until CT in NAAT+ve group is shown by the colour gradient. c) Dot plots showing sRNA feature expression across time points separated by vaccine group. The whiskers mark the Q1 – 1.5*IQR and Q3 + 1.5*IQR. D0 n=19, CT NAAT+ve ChAdOx1 nCoV-19 n=21, CT NAAT+ve placebo n=30, CT+7 NAAT+ve ChAdOx1 nCoV-19 n=21, CT+7 NAAT+ve placebo n=31. Differential expression analysis was performed using a two-sided moderate t-test.

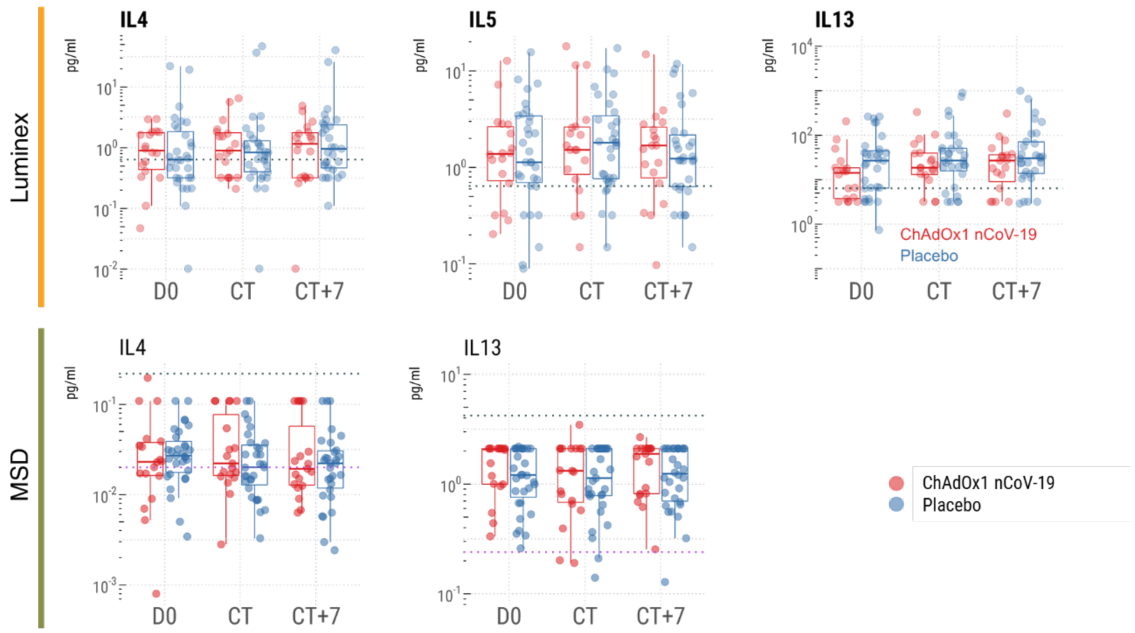

Supplementary figure 6: Th2 cytokines measured prior to and during COVID-19 episode on Luminex and MSD. Each dot represents a volunteer, displayed with medians and IQRs. The limit of detection is indicated by a dotted line on Luminex graphs (top row), and values below the limit of detection were assigned a value of half the limit of detection. The black dotted line indicates the Lower limit of Quantification, and the purple dotted line indicates the Lower limit of Detection on MSD graphs (bottom row). Statistical comparisons were applied among the 2 groups (ChAdOx1 nCoV-19 vaccinees – red and placebo – blue) at each time point by unpaired two-sided Wilcoxon test with FDR test adjustment. D0 ChAdOx1 nCoV-19 n=18, D0 placebo n=31, CT ChAdOx1 nCoV-19 n=19, CT placebo n=31, CT+7 ChAdOx1 nCoV-19 n=19, CT+7 placebo n=30.

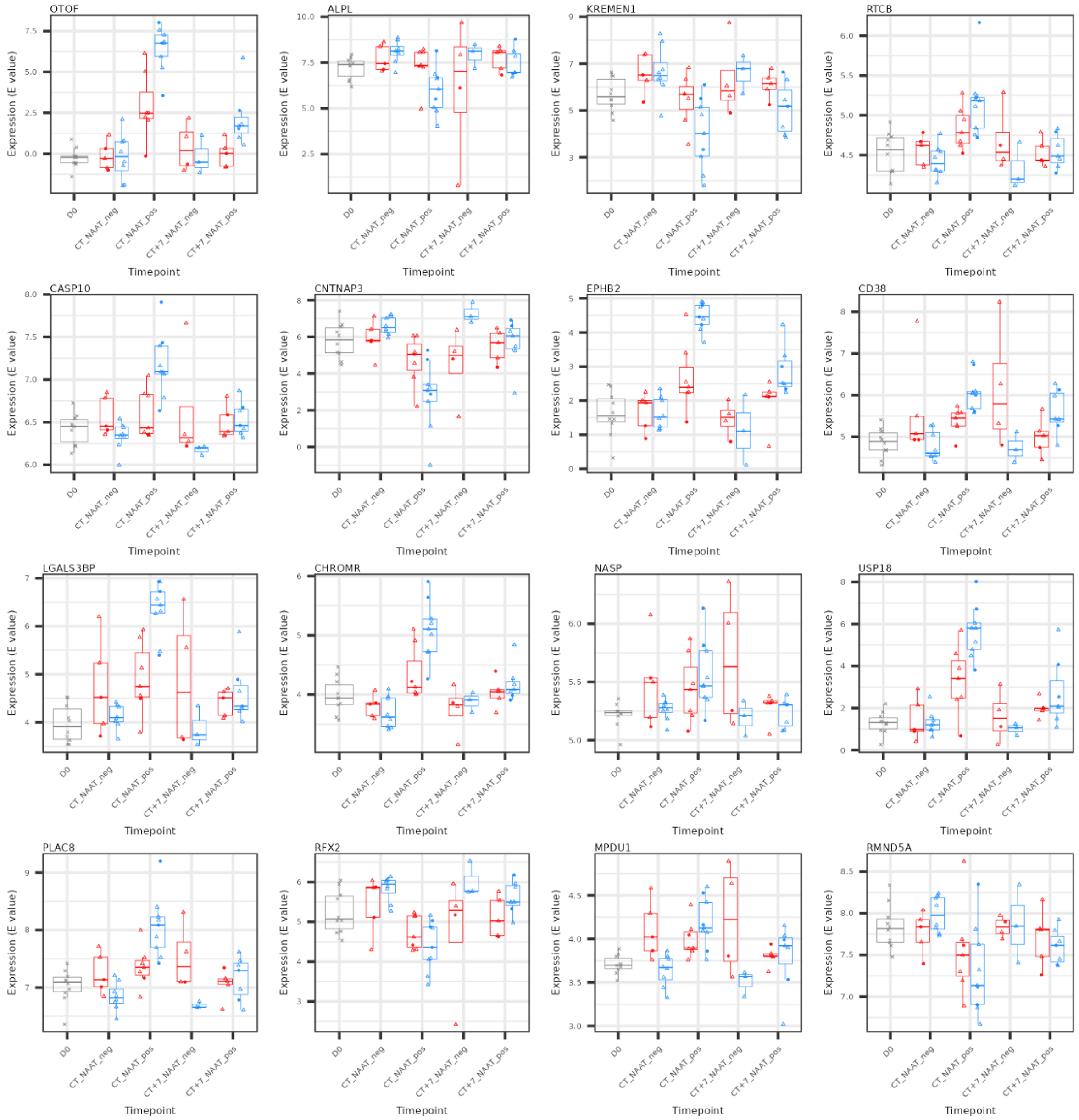

Supplementary figure 7: Dot plots of stage 1 data displaying the expression levels of the 16 most differentially expressed genes between vaccine groups at COVID-19 symptom onset in the stage 2 study dataset. D0 n=10, CT NAAT-ve ChAdOx1 nCoV-19 n=5, CT NAAT-ve placebo n=8, CT NAAT+ve ChAdOx1 nCoV-19 n=7, CT NAAT+ve placebo n=9, CT+7 NAAT-ve ChAdOx1 nCoV-19 n=3, CT+7 NAAT-ve placebo n=4, CT+7 NAAT+ve ChAdOx1 nCoV-19 n=5, CT+7 NAAT+ve placebo n=7. The whiskers mark the Q1 – 1.5*IQR and Q3 + 1.5*IQR.

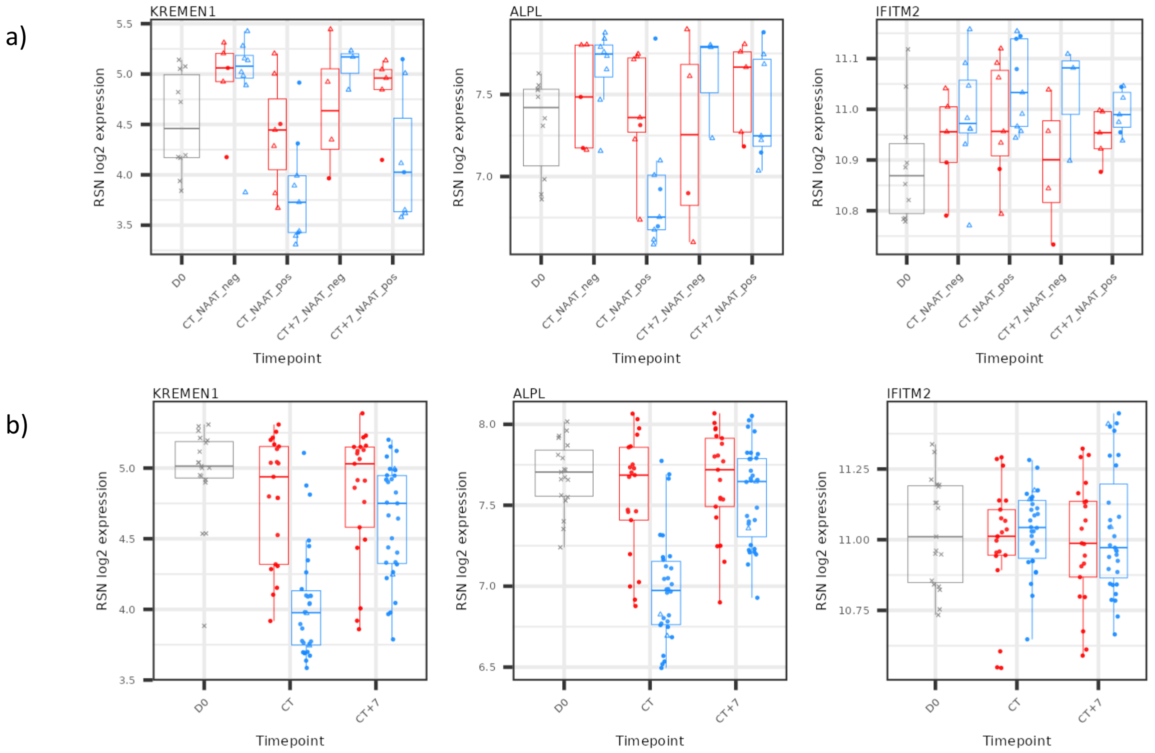

Supplementary figure 8: Dot plots displaying selected imputed gene expression levels in neutrophils from CibersortX analysis for a) stage 1, D0 n=10, CT NAAT-ve ChAdOx1 nCoV-19 n=5, CT NAAT-ve placebo n=8, CT NAAT+ve ChAdOx1 nCoV-19 n=7, CT NAAT+ve placebo n=9, CT+7 NAAT-ve ChAdOx1 nCoV-19 n=3, CT+7 NAAT-ve placebo n=4, CT+7 NAAT+ve ChAdOx1 nCoV-19 n=5, CT+7 NAAT+ve placebo n=7; and b) stage 2, D0 n=19, CT NAAT+ve ChAdOx1 nCoV-19 n=21, CT NAAT+ve placebo n=30, CT+7 NAAT+ve ChAdOx1 nCoV-19 n=21, CT+7 NAAT+ve placebo n=31. The whiskers mark the Q1 – 1.5*IQR and Q3 + 1.5*IQR.

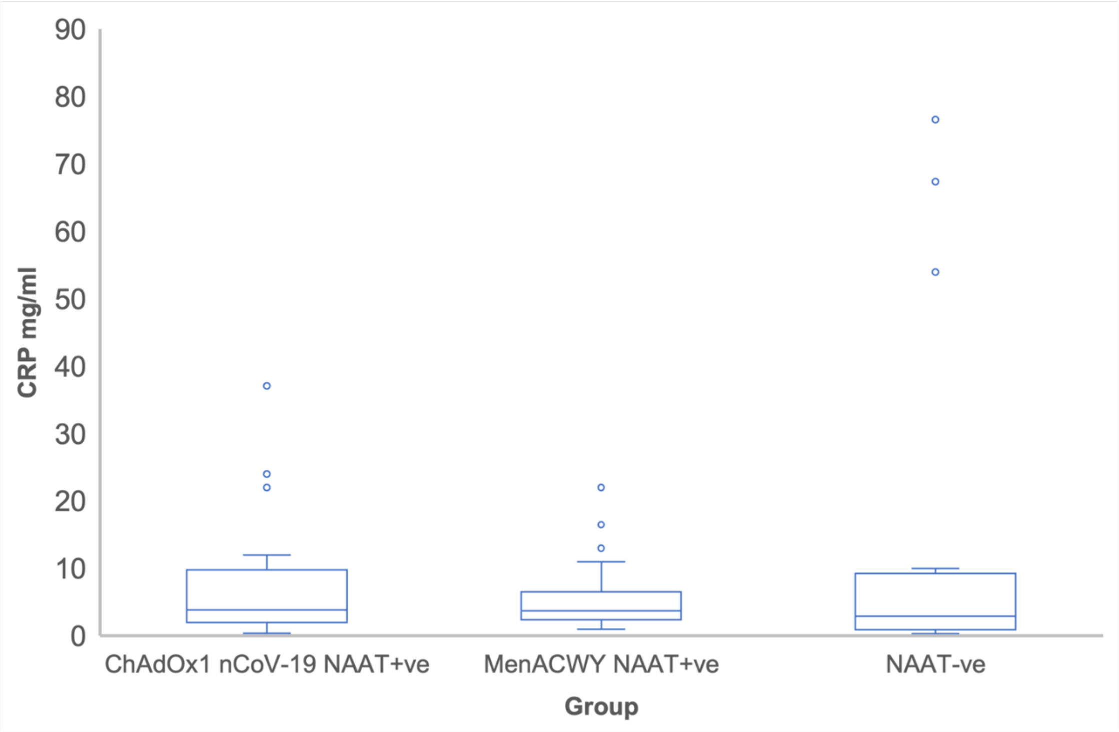

Supplementary figure 9: CRP results at CT for Stage 1 and Stage 2 combined. ChAdOx1 NAAT+ve n=28; placebo NAAT+ve n=40, NAAT-ve n=18. The whiskers mark the Q1 – 1.5*IQR and Q3 + 1.5*IQR.

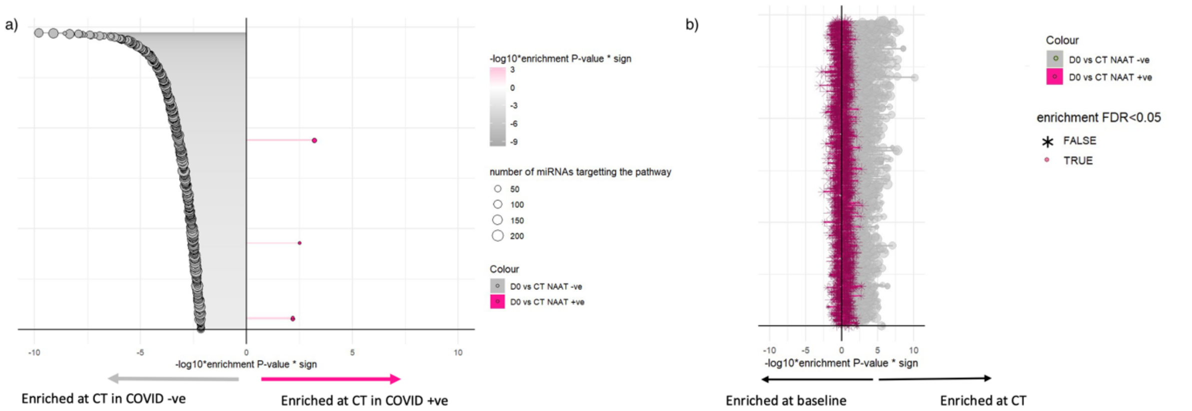

Supplementary figure 10: Stage 1 data indicated increased miRNA targeting of immune pathways in NAAT-ve participants compared with participants with COVID-19. a) MIEAA pathway enrichment output using the results of the miRNA expression analysis comparing CT NAAT-ve (n=13) versus CT NAAT+ve (n=16) ranked by t statistic. Significance testing obtained via MIEAA which uses a GSEA analysis approach. Only results with FDR < 0.05 are shown. Pathways which are more enriched for targeting at CT in the NAAT-ve group are coloured grey and plot towards the left. Pathways which are more enriched for targeting at CT in the NAAT-ve group are coloured pink and plot towards the right. b) For the pathways shown in a), target enrichment results from MIEAA2 using the results (ranked by t statistic) of baseline (D0, n=10) versus CT NAAT-ve (grey, n=13) and baseline versus CT NAAT+ve (pink, n=16). Pathways which are more enriched for targeting at baseline plot to the left. Pathways which are more enriched for targeting at CT plot towards the right. Pathways are plotted irrespective of whether they were FDR significant. Significance testing obtained via MIEAA which uses a GSEA analysis approach.

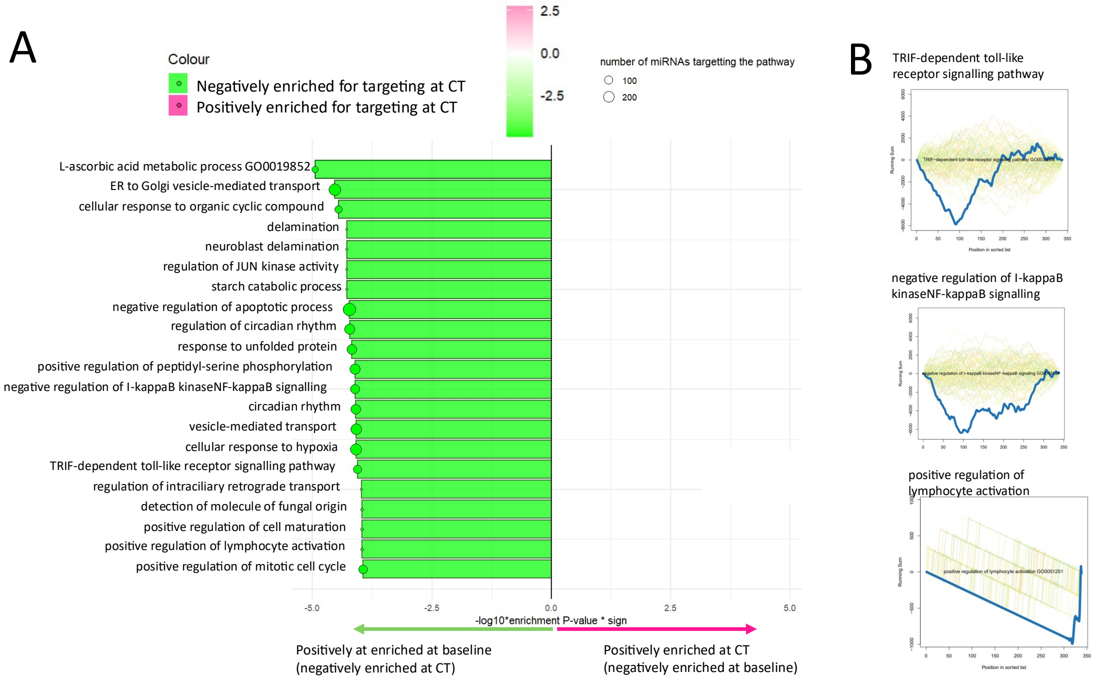

Supplementary figure 11: Stage 2 data: Gene ontology pathways enriched at CT compared with baseline. a) Enrichment analysis results comparing symptom onset in COVID-positive and baseline. Enrichment results presented for significantly enriched pathways based on miRNA expression levels comparing NAAT+ve CT (n=1) with baseline (D0, n=19). Each dot represents the result for a different pathway. Only pathways enriched at an FDR<0.05 are shown. The MIEA2 software does not return enrichment scores; therefore, the -$\log_{10}$ of the p-values are presented alongside the direction of enrichment indicated. Pathways positively enriched for targetting (i.e. pathways predicted to be subject to greater miRNA expression) at baseline (i.e. negatively enriched in the COVID-19 positive group) plot towards the left and are coloured green. Pathways positively enriched for targetting in the COVID-19 positive group plot towards the right and are coloured pink. The magnitude of enrichment is represented by the distance from 0 on the x-xis and the intensity of the colour of the dots and bar. The plot shows that all pathways were more enriched for targetting at baseline, indicating that miRNAs limit pathway expression during health. Significance testing obtained via MIEAA which uses a GSEA analysis approach. B) Examples of enrichment plots for three pathways are shown in plot a).

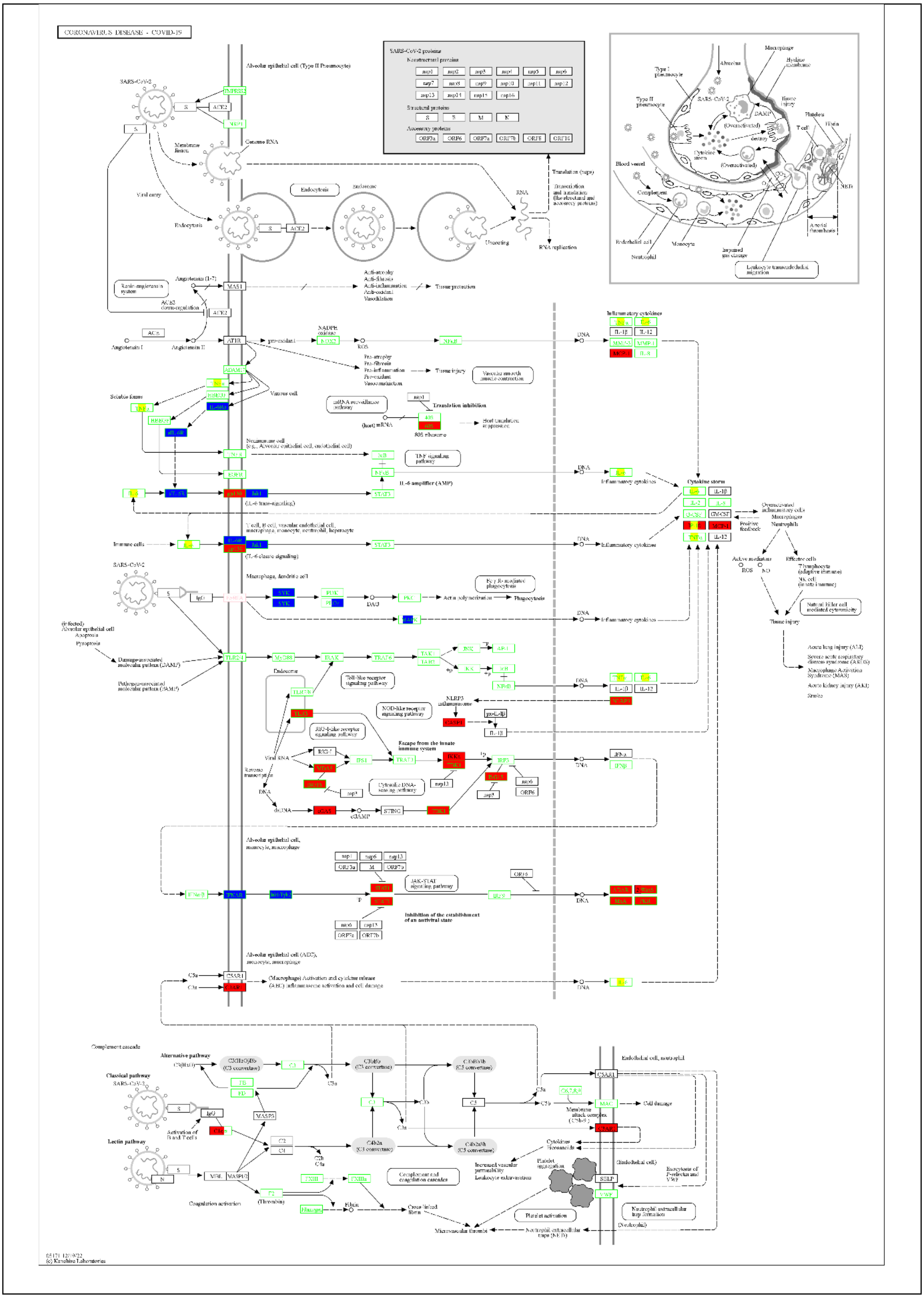

Supplementary figure 12: COVID-19 DISEASE KEGG pathway with differentially expressed features highlighted. Differentially expressed features are those that were identified in the baseline vs CT NAAT+ve analyses in this manuscript. Green outline and text = targets of differentially expressed miRNA, red fill = upregulated at the mRNA level, blue fill = downregulated at the mRNA level, internal yellow stripe = upregulated in serum at the protein level.

Pink outline – an sRNA that lies reverse complement to FCGR2 (an antibody FC receptor) was downregulated. Pathway was rendered via the KEGG pathway database accessible at https://www.genome.jp/kegg/pathway.html.

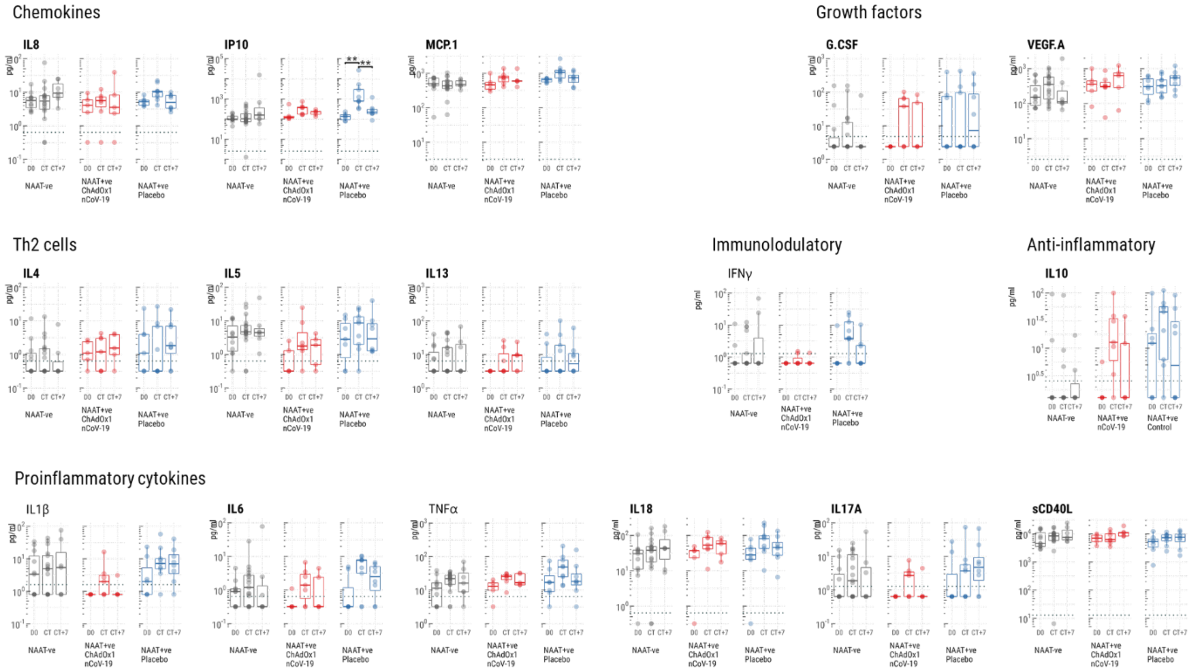

Supplementary figure 13: Trend of serum cytokine concentrations on time at Stage 1. Levels (pg/ml) of 16 detectable cytokines (Chemokines IL8, IP10 and MCP1, growth factors G.CSF and VEGF.A, type 2 cytokines IL4, IL5 and IL13, immunomodulatory IFNγ, anti-inflammatory cytokine IL10 and pro-inflammatory cytokines IL1β, IL6, TNFα, IL18, IL17A and sCD40L) measured by Luminex on Stage1 before vaccinations (D0), around symptom onset (CT) and 7 days later (CT+7) in NAAT-ve (grey, D0 n=11, CT n=17, CT+7 n=7), NAAT+ve receiving ChAdOx1 nCoV-19 vaccine (red, D0 n=5, CT n=7, CT+7 n=5) and NAAT+ve who received the placebo vaccine (blue, D0 n=7, CT n=9, CT+7 n=8). Each dot represents a volunteer, displayed with medians and IQRs. The dotted line indicates the limit of detection, and values below the limit of detection were assigned a value of half the limit of detection. Statistical comparisons were applied among the 3 groups at each time point by two-sided unpaired Wilcoxon test with FDR test for adjusting, shown as *FDR < 0.05, **FDR < 0.01, ***FDR < 0.001.

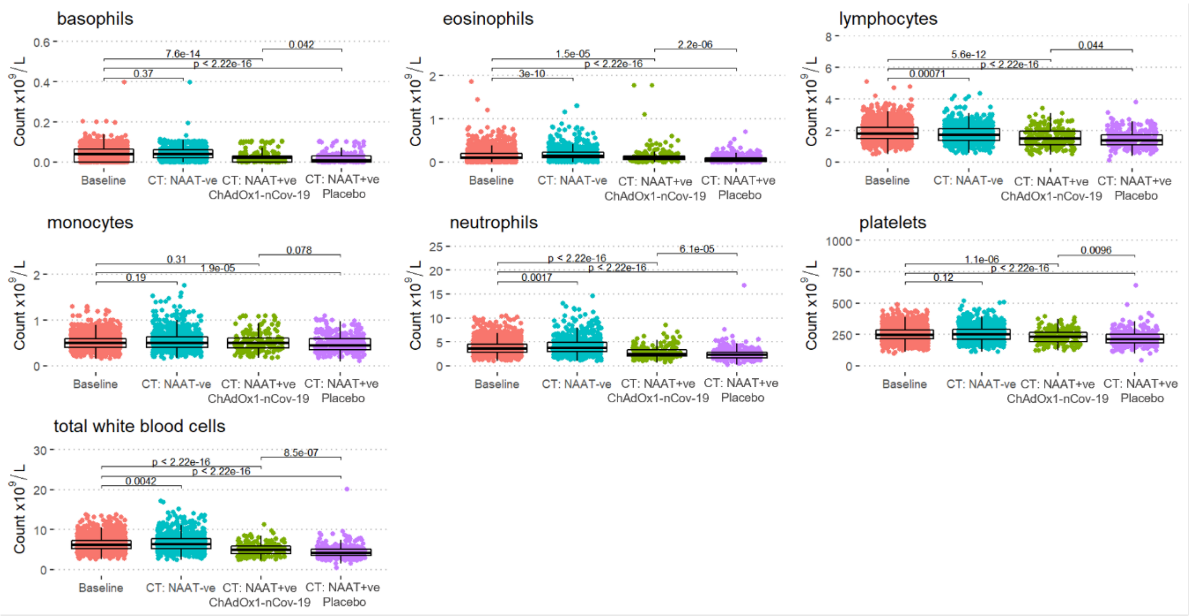

Supplementary figure 14: Full blood count breakdown at baseline and CT across groups measured by clinical complete bloods counts. Baseline, D0 n=2276, CT NAAT-ve n=835, CT NAAT+ve ChAdOx1 nCoV-19 n=190, CT NAAT+ve placebo n=327. Each dot represents a volunteer. The centre line denotes the median value (50th percentile, Q2), the box contains the 25th (Q1) to 75th (Q3) percentiles of dataset. The whiskers mark the Q1 – 1.5*IQR and Q3 + 1.5*IQR. P-values were derived from two-sided Wilcoxon rank-sum tests. Source data are provided as a Source Data file.

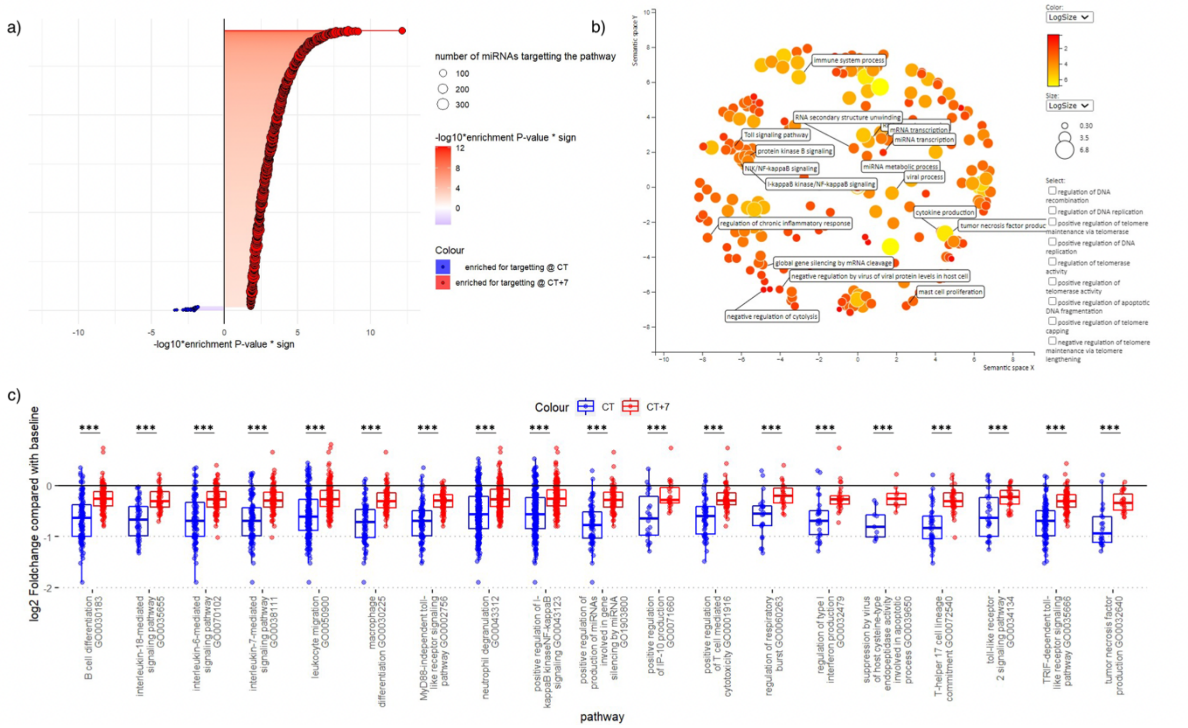

Supplementary figure 15: Restoration of miRNA expression 7 days after COVID-19 onset reimposes post transcriptional regulation of immune pathways. Stage 2 data. a) miRNA pathway enrichment results for CT vs CT+7 showing enriched GO:BP terms. Significance testing obtained via MIEAA which uses a GSEA analysis approach. b)

REVIGO summary of significantly enriched GO:BP. c) Examples of log_2 foldchanges of miRNAs that target genes in CT+7 enriched immune-related pathways shown in a), CT NAAT+ve n=51, CT+7 NAAT+ve n=52.

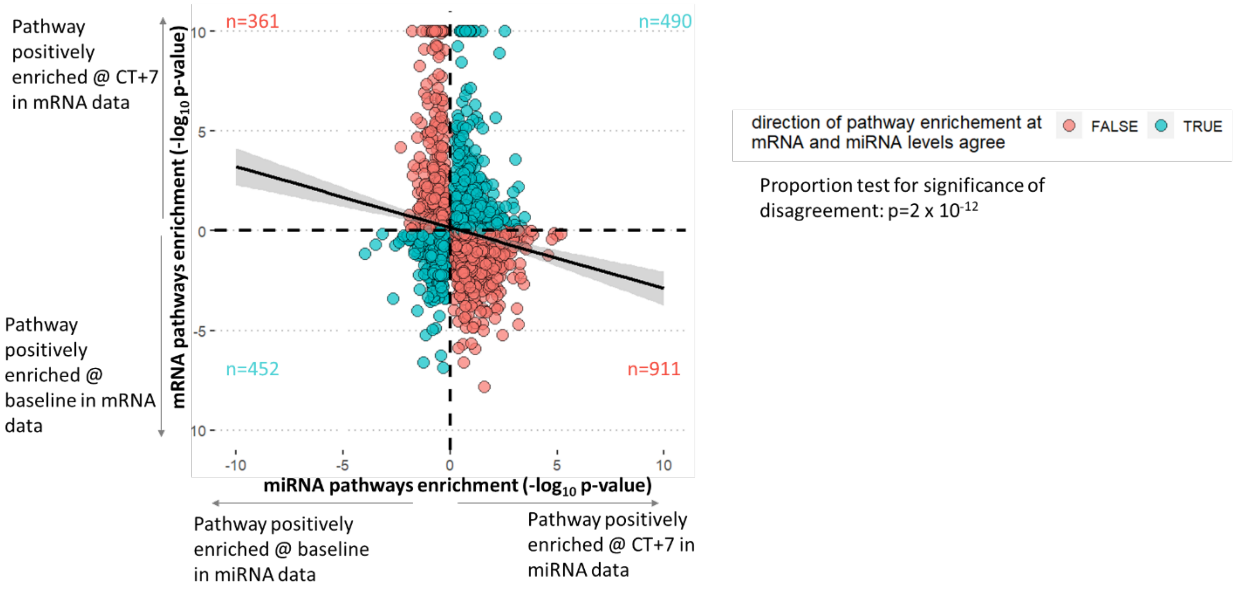

Supplementary figure 16: Stage 2 data. Agreement plot for GO:BP enrichment in the mRNA and miRNA data. Each dot represents the results of a pathway. The x-axis shows the log1o of the p-value of the pathway enrichment in the miRNA data. The y-axis shows the log_{10} of the p-value of the pathway enrichment in the mRNA data multiplied by the enrichment sign (+1 if positive, -1 if negative). The colour indicates whether the sign of miRNA enrichment and mRNA enrichment results for a pathway agree (blue) or disagree (red). This can also be gauged from the quadrants of the graph the pathway lies in. The numbers in each quadrant show how many pathways fall in that quadrant. The results for all pathways tested in the miRNA and mRNA data are shown. Spearman rank r= - 0.14, p=1.38x10^{-11}.

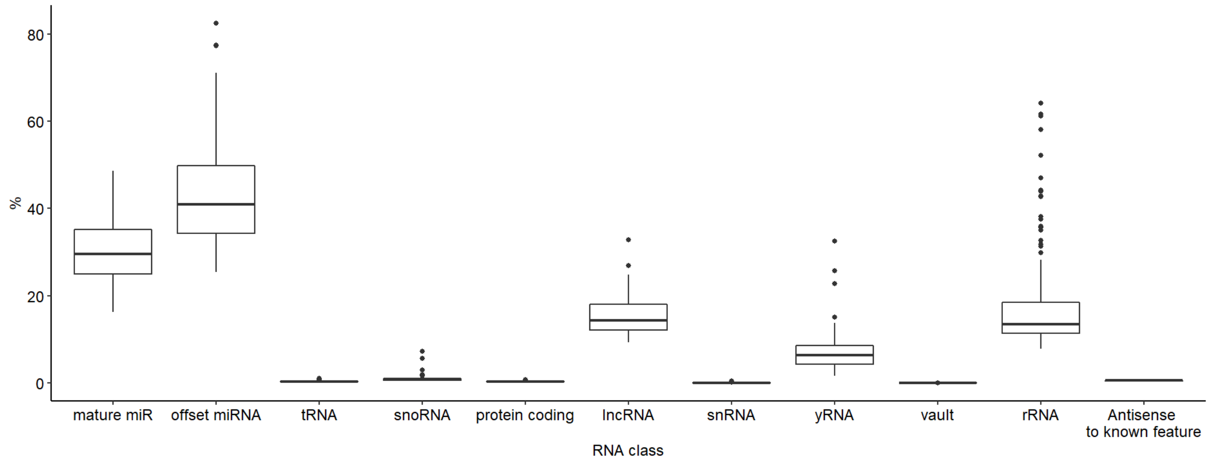

Supplementary figure 17: Stage 2 Relative abundance of each sRNA class captured by sRNA sequencing in stage 2. Each boxplot summarises the total expression of each RNA class across samples (n=122). The whiskers mark the Q1 – 1.5*IQR and Q3 + 1.5*IQR.

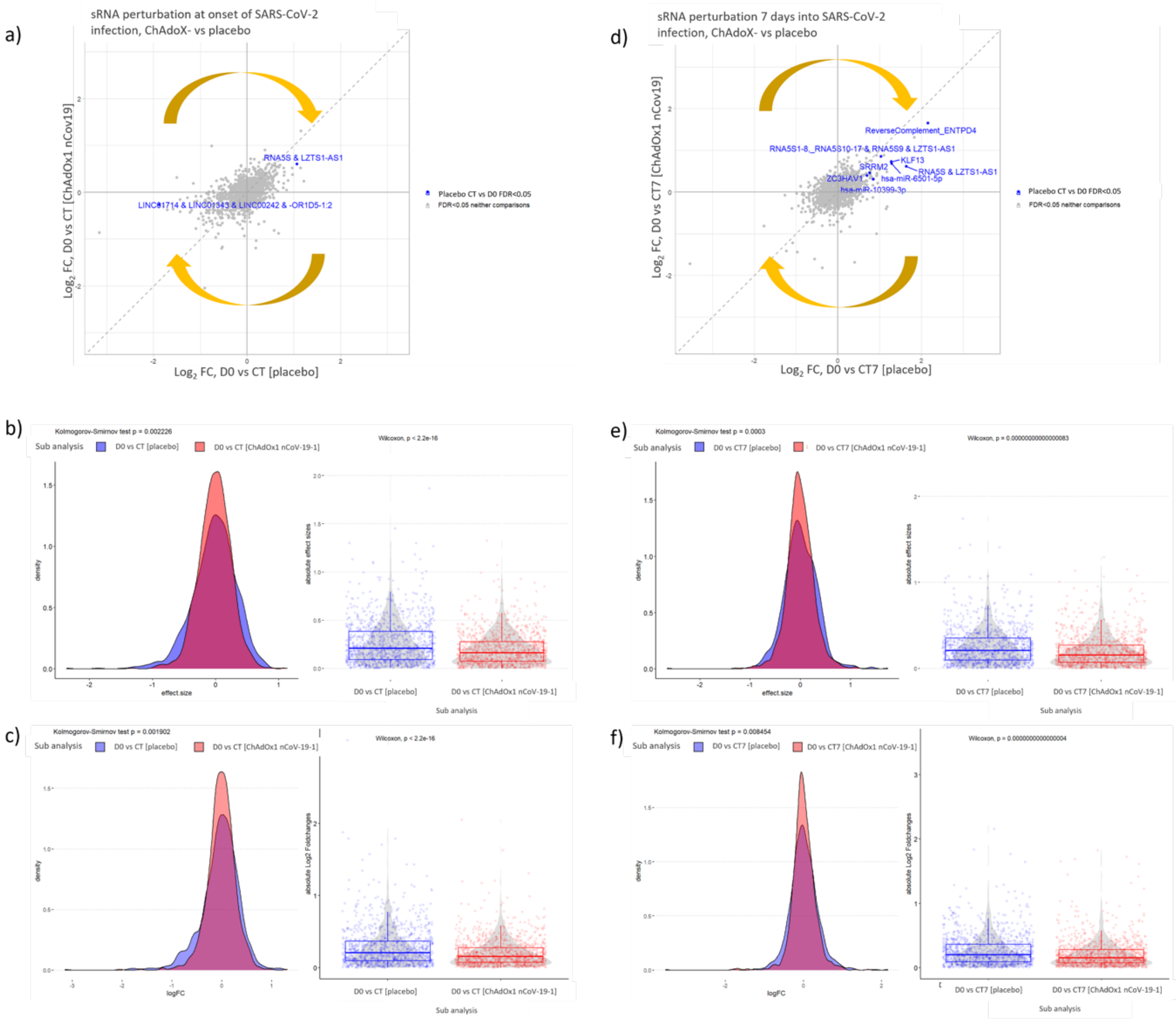

Supplementary figure 18: Stage 1 data shows ChAdOx1 nCoV-19 attenuates sRNA change at COVID-19 onset and 7 days later. a) Agreement plot of differentially expressed genes at NAAT+ve CT vs baseline in Placebo (x-axis, n=9) and ChAdOx1 nCoV-19 (y-axis, n=7) vaccine recipients compared with baseline (D0, n=10) in the sRNA. Yellow arrows highlight direction of changes observed compared to baseline. b) Comparing the distribution of effect sizes (left) and box plot of absolute effect sizes (right) between NAAT+ve CT vs baseline (D0, n=10) in Placebo (blue, n=9) and ChAdOx1 nCoV-19 (red, n=7) vaccine recipients in the sRNA data. Significance values comparing the distribution (two-sided Kolmogorov-Smirnov) and average absolute effect size between the vaccine groups shown on plots. c) Comparing the distribution of log_2 foldchange (left) and box plot of absolute log_2 foldchange (right) between NAAT+ve CT vs baseline (D0, n=10) in Placebo (blue, n=9) and ChAdOx1 nCoV-19 (red, n=7) vaccine recipients in the sRNA data. Significance values comparing the distribution (two-sided Kolmogorov-Smirnov) and average absolute log_2 foldchange between the vaccine groups shown on plots. d) Agreement plot of differentially expressed genes at NAAT+ve CT+*7* vs baseline in Placebo (x-axis, n=*7*) and ChAdOx1 nCoV-19 (y-axis, n=*5*) vaccine recipients compared with baseline (D0, n=10) in the sRNA. Yellow arrows highlight direction of changes observed compared to baseline. *e)* Comparing the distribution of effect sizes (left) and box plot of absolute effect sizes (right) between NAAT+ve CT+*7* vs baseline (D0, n=10) in Placebo (blue, n=*7*) and ChAdOx1 nCoV-19 (red, n=*5*) vaccine recipients in the sRNA data. Significance values comparing the distribution (two-sided Kolmogorov-Smirnov) and average absolute effect size between the vaccine groups shown on plots. *f)* Comparing the distribution of log_2 foldchange (left) and box plot of absolute log_2 foldchange (right) between NAAT+ve CT+*7* vs baseline (D0, n=10) in Placebo (blue, n=*7*) and ChAdOx1 nCoV-19 (red, n=*5*) vaccine recipients in the sRNA data. Significance values comparing the distribution (two-sided Kolmogorov-Smirnov) and average absolute log_2 foldchange between the vaccine groups shown on plots.

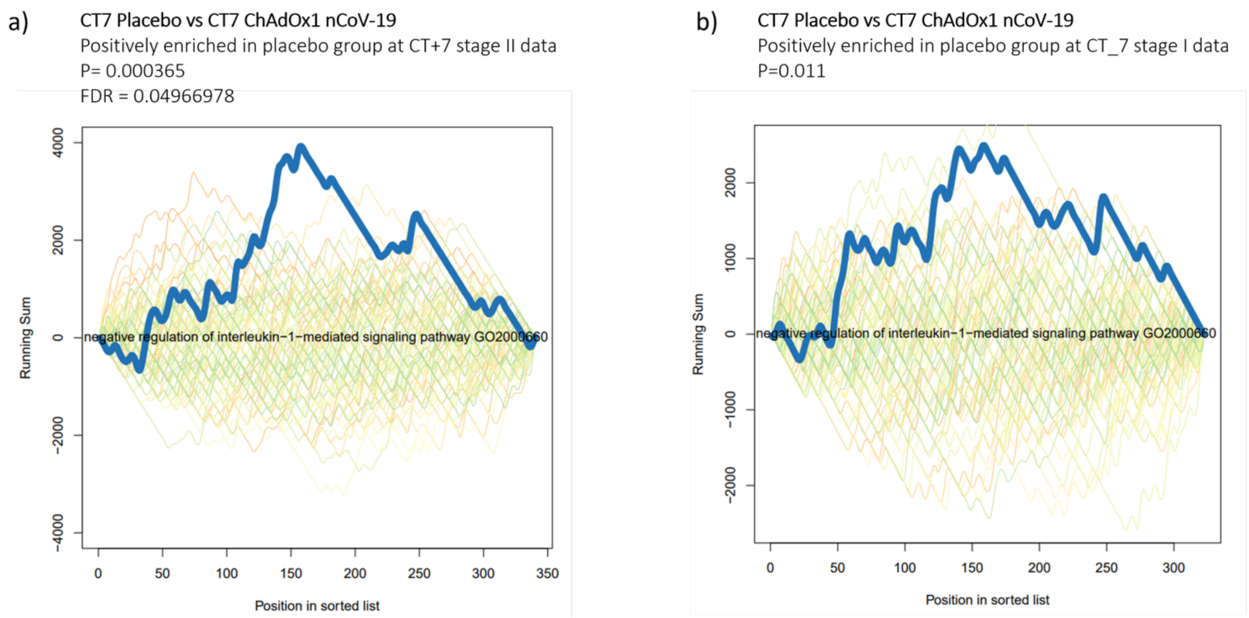

Supplementary figure 19: Example of a GO:BP pathway differentially enriched between placebo and ChAdOx1 nCoV-19 groups in stage 2 and confirmed in stage 1 data. Running sum of the "negative regulation of interleukin-1-mediated signalling pathway" derived from MIEAA2 GSEA style analysis in a) the stage 2 data (CT+7 ChAdOx1 nCoV-19 n=21, CT+7 placebo n=31) and b) stage 1 data (CT+7 ChAdOx1 nCoV-19 n=5, CT+7 placebo n=7). Figure downloaded from MIEA website: https://www.ccb.uni-saarland.de/mieaa2. miRNAs inputted into GSEA analysis ranked by t-statistic ranked from most upregulated at CT+7 in the placebo vs ChAdOx1 nCoV-19 group to most downregulated at CT+7 in the placebo vs ChAdOx1 nCoV-19 group.

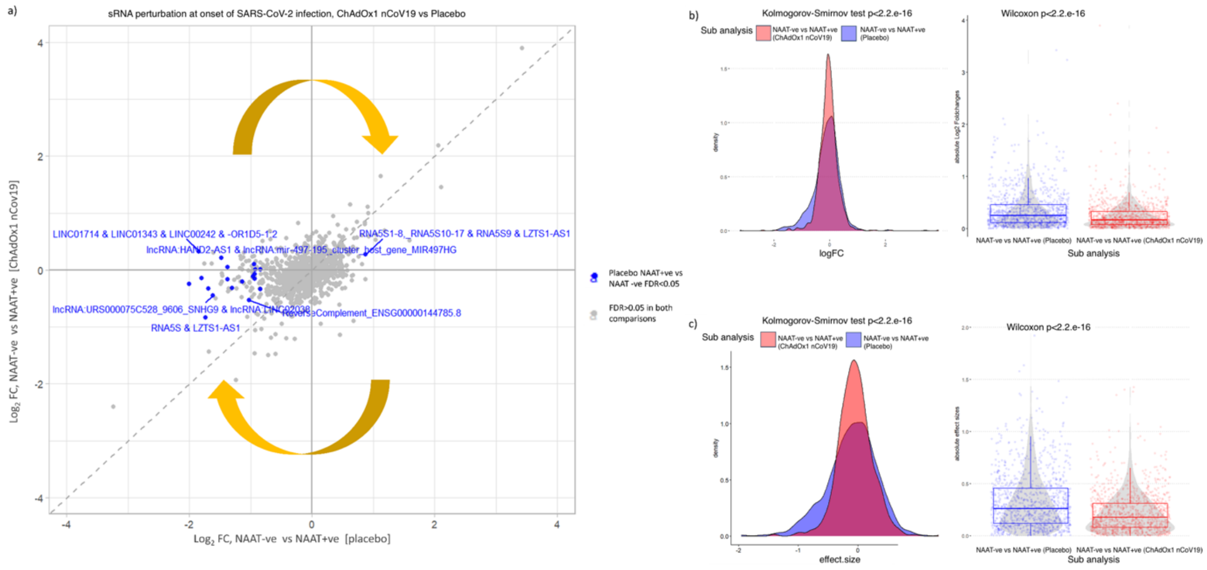

Supplementary figure 20: More profound changes in sRNA expression in COVID-19 in placebo compared with ChAdOx1 nCoV-19 vaccinees. a) Agreement plot of differentially expressed genes at CT NAAT+ve vs CT NAAT-ve in Placebo (x-axis, CT NAAT-ve n=8, CT NAAT+ve n=9) and ChAdOx1 nCoV-19 (y-axis, CT NAAT-ve n=5, CT NAAT+ve n=7) vaccine recipients in the sRNA data. Yellow circular lines represent the direction of changes observed in NAAT+ve compared to NAAT-ve. b) Stage 1 data. Comparing the distribution *of log2 foldchange* (left) and box plot of *absolute* log_2 *foldchange* (right) between CT NAAT+ve vs CT NAAT-ve in Placebo (blue, CT NAAT-ve n=8, CT NAAT+ve n=9) and ChAdOx1 nCoV-19 (red, CT NAAT-ve n=5, CT NAAT+ve n=7) vaccine recipients in the sRNA data. Significance values

comparing the distribution (two-sided Kolmogorov-Smirnov) and average *log2 foldchange* between the vaccine groups shown on plots. c) Stage 1 data. Comparing the distribution of *effect sizes (left*) and box plot of *absolute effect sizes* (right) between NAAT+ve CT vs NAAT-ve CT in Placebo (blue, CT NAAT-ve n=8, CT NAAT+ve n=9) and ChAdOx1 nCoV-19 (red, CT NAAT-ve n=5, CT NAAT+ve n=7) vaccine recipients in the sRNA data. Significance values comparing the distribution (Kolmogorov-Smirnov) and average absolute effect size between the vaccine groups given at tops of plots.

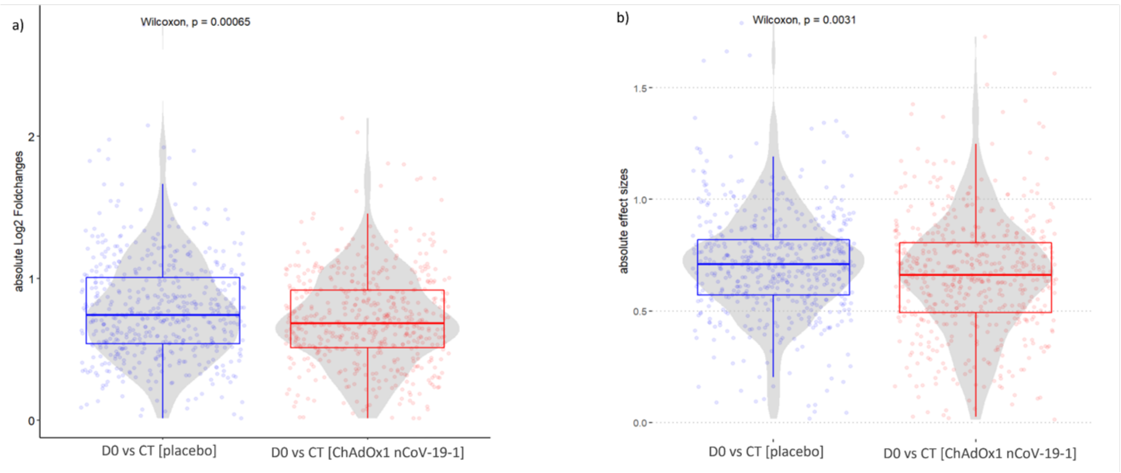

Supplementary figure 21: Stage 2 data shows ChAdOx1 nCoV-19 attenuates sRNA change at COVID-19 onset a) Comparing absolute log_2 foldchange in between NAAT+ve CT vs baseline (D0, n=19) in individuals in Placebo (blue, n=30) and ChAdOx1 nCoV-19 (red, n=21) vaccine recipients in the sRNA data for genes differentially expressed (FDR< 0.05) in at least one of the groups compared with baseline. Significance values comparing the and average absolute effect size between the vaccine groups given at to of plot. b) Comparing absolute effect sizes between NAAT+ve CT vs baseline (D0, n=19) in individuals in Placebo (blue, n=30) and ChAdOx1 nCoV-19 (red, n=21) vaccine recipients in the sRNA data for genes differentially expressed (FDR< 0.05) in at least one of the groups compared with baseline. Significance value comparing the and average absolute effect size between the vaccine groups given at top of plot – two-sided Wilcoxon test used.

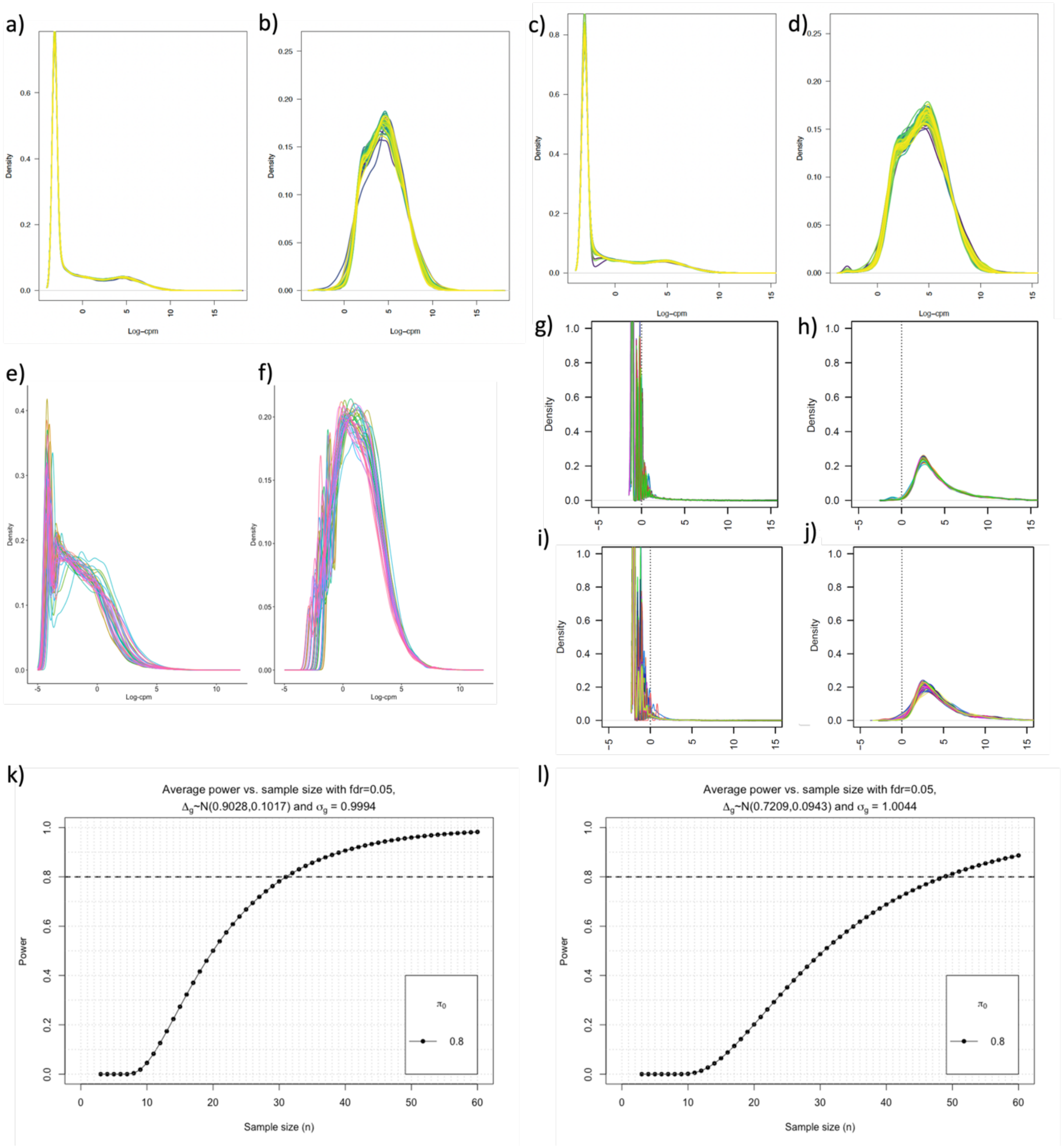

Supplementary figure 22: Density plots before and after filtering of the features, each line represents a single sample. a) Illumina nex-gen RNA-sequencing, stage 1 data before filtering (n=58 samples). b) Illumina nex-gen RNA-sequencing, stage 1 data after filtering (n=58 samples). c) Illumina nex-gen RNA-sequencing, stage 2 data before filtering (n=122 samples). D) Illumina nex-gen RNA-sequencing, stage 2 data after filtering (n=122 samples). e) ONT 3rd-gen RNA sequencing, stage 1 data before filtering (n=39 samples). f) ONT 3rd-gen RNA sequencing, stage 1 data after filtering (n=39 samples). g) Small RNA-sequencing, stage 1 data before filtering (n=58 samples). h) Small RNA-sequencing, stage 1 data after filtering (n=58 samples). I) Small RNA-sequencing, stage 2 data before filtering (n=122 samples). J) Small RNA-sequencing, stage 2 data after filtering (n=122 samples). k) Average power vs sample size for stage 1 cohort (n=58 samples) for next-gen RNA seq. l) Average power vs sample size for stage 1 cohort (n=58 samples) for small RNA seq. Derived using the ssize package in R.

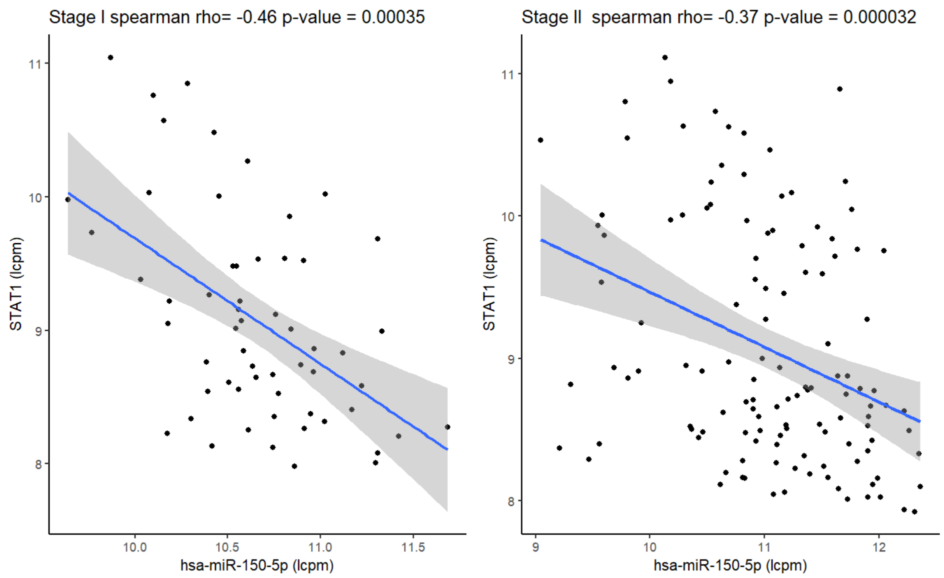

Supplementary figure 23: Correlation between the expression of hsa-miR-150-5p and STAT1 - known experimentally validated target pairs. In the stage 2 cohort, STAT1 and hsa-miR-150-5p were up and downregulated respectively between baseline and CT at FDR<0.05. Blue line is line of best fit, grey shading represents 95% confidence intervals of line of best fit.

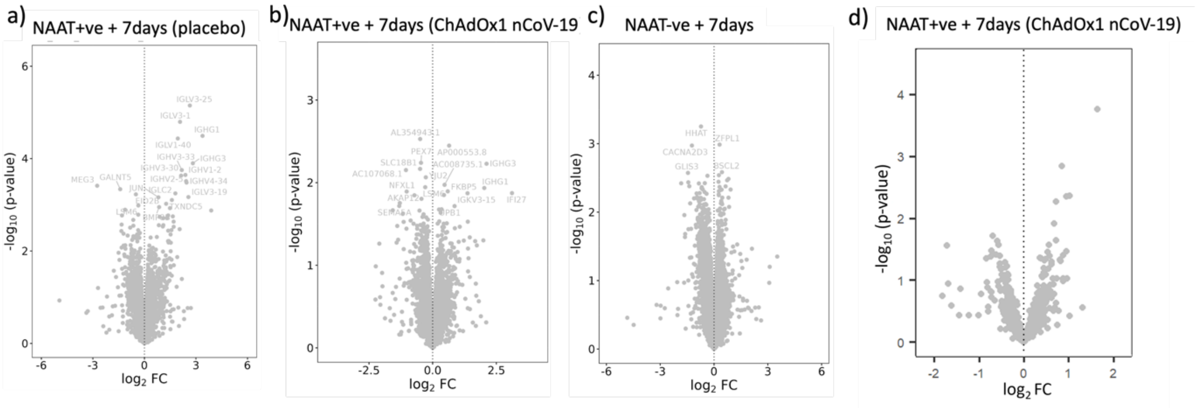

Supplementary figure 24: Stage 1 data showed no differences in gene expression 7 days after COVID-19 onset when compared with health or covid-like illness a) Volcano plot of next-gen RNA-seq gene expression at CT+7 in NAAT+ve placebo vaccinated individuals (n=7) compared with baseline (D0, n=10) samples. b) Volcano plot of gene expression at CT+7 consistent with COVID-19 in NAAT+ve (ChAdOx1 nCoV-191 nCoV-19, n=5) individuals compared with baseline (D0, n=10) samples. c) Volcano plot of gene expression at CT+7 in NAAT-ve individuals (n=7) compared with baseline (D0, n=10) samples. d) Volcano plot comparing the small RNA-seq blood transcriptome at CT+7 in NAAT+ve (ChAdOx1 nCoV-19, n=5) individuals compared with baseline (D0, n=10) samples. Differential expression analysis was performed using a two-sided moderate t-test.

Supplementary Tables

Supplementary Table 1 – Illness severity assessment and classification criteria

Severity of illness	Mild	Moderate A	Moderate B	Severe
Features	- Completing full sentences<br>- No SOB<br>- No chest tightness<br>- Able to do ADLs<br>- No other red flags/ concerning features from history & examination<br>Observations:<br>- RR 12-20<br>- HR 50-100<br>- SpO2≥95%	- Completing full sentences<br>- Able to do ADLs but lethargic<br>- Mild chest tightness<br>- Mild SOB on exertion only<br>- No other red flags/ concerning features from history & examination<br>- Any symptoms from other systems considered to be moderate and not requiring medical review<br>Observations:<br>- RR 12-20<br>- HR 50-100<br>- SpO2≥95%	- Completing full sentences<br>- Able to do ADLs but lethargic<br>- Mild chest tightness<br>- Mild SOB on exertion<br>- Any symptoms from other systems considered to be moderate and requiring medical review<br>Observations (any one of the following automatically classifies as Moderate B):<br>- RR 20-24<br>- HR persistently 100-130<br>- SpO2 93-94%	Any one of the following:<br>- Inability to complete full sentences<br>- Unable to do any ADLs/ get out of bed<br>- Any other clinical concerns for severe disease in any system e.g. cyanosis/ confusion<br>Observations:<br>- RR>25<br>- HR >130<br>- SpO2≤92%

*SOB - Shortness Of Breath; ADLs - Activities of Daily living; RR - Respiratory Rate; HR – Heart Rate; SpO2 - Oxygen Saturations

Supplementary Table 2 – Average RNA-seq statistics

Median number of reads obtained via RNA-sequencing

Data type	Stage 1 (IQR)	Stage 2 (IQR)
Illumina next-gen RNA sequencing	49,480,184 (47,059,636-52,301,527)	39,389,562 (38,644,940-40122655)
ONT 3rd gen RNA sequencing	68,773,129 (61,366,383-73,932,474)	N/A
sRNA	4,440,745 (3,850,706-,962,543)	8,226,562 (7,762,793-9,738,660)

Median number of reads mapped to analysed features

Data type	Stage 1 (IQR)	Stage 2 (IQR)
Illumina next-gen RNA sequencing	45,432,354 (43,341,599-48,168,246)	35,856,747 (35,130,346-36,485,071)
ONT 3rd gen RNA sequencing	65,064,582 (58,155,344-70,182,215)	N/A

sRNA	4,327,768 (3,761,417-4,850,598)	7,996,464 (7,512,018-9,494,672)

3rd gen RNA sequencing full statistics

Participant number	timepoint	Vaccine	NAAT result	Reads	Mapped	Read length (avg)	Read length (N50)
1	D0	ChAdOx1 nCoV-19	1	61,223,233	59,336,400	773.6	794
1	CT	ChAdOx1 nCoV-19	1	61,509,533	56,974,288	822.3	826
2	D0	MenACWY	1	71,529,753	69,395,193	769.4	795
2	CT	MenACWY	1	66,865,545	61,294,901	754.3	788
3	D0	ChAdOx1 nCoV-19	0	80,484,979	76,185,834	774.5	798
3	CT	ChAdOx1 nCoV-19	0	74,618,760	70,753,402	758.6	790
4	D0	MenACWY	0	63,757,599	61,411,078	795.3	807
4	CT	MenACWY	0	70,627,104	67,958,985	796.2	801
5	D0	MenACWY	0	58,443,341	55,884,907	762.9	801
5	CT	MenACWY	0	68,773,129	65,770,631	760.2	794
6	CT	ChAdOx1 nCoV-19	0	69,041,764	61,783,915	764.8	797
7	D0	MenACWY	0	54,726,104	52,497,313	793.8	796
7	CT	MenACWY	0	82,019,086	77,922,835	765.7	790
8	D0	ChAdOx1 nCoV-19	1	69,423,042	64,450,151	747.2	790
8	CT	ChAdOx1 nCoV-19	1	73,712,350	70,947,992	777.9	790
10	D0	MenACWY	0	69,652,518	67,461,318	778.7	795
10	CT	MenACWY	0	74,152,597	71,595,250	778.5	797
11	CT	MenACWY	0	83,563,860	76,533,140	719.1	792
12	CT	ChAdOx1 nCoV-19	0	70,656,550	67,859,528	762.9	794
13	D0	ChAdOx1 nCoV-19	0	65,356,356	62,585,662	770.2	797
13	CT	ChAdOx1 nCoV-19	0	78,411,489	74,987,279	798.4	804
14	D0	ChAdOx1 nCoV-19	0	59,535,378	53,493,845	656.6	779
14	CT	ChAdOx1 nCoV-19	0	46,098,368	42,667,732	734.8	794
15	CT	ChAdOx1 nCoV-19	1	60,167,407	56,291,091	809.3	802
16	CT	ChAdOx1 nCoV-19	1	52,605,013	49,110,258	838.8	829
17	CT	ChAdOx1 nCoV-19	1	69,976,056	67,089,638	786.6	791
18	CT	MenACWY	0	76,646,832	72,037,548	768.3	794
19	CT	MenACWY	0	67,503,893	65,153,703	762.7	785
20	CT	MenACWY	0	79,254,110	74,444,093	782.7	791
21	CT	MenACWY	1	55,802,655	52,079,010	843.6	818
22	CT	MenACWY	1	75,142,851	69,611,028	747.5	802
23	CT	MenACWY	1	77,034,672	73,609,061	749.6	785

24	CT	ChAdOx1 nCoV-19	1	63,258,789	60,330,308	798.9	796
25	CT	MenACWY	1	56,300,114	52,577,416	894.9	841
27	CT	ChAdOx1 nCoV-19	1	73,638,249	69,354,953	752.6	796
28	CT	MenACWY	1	67,335,178	63,159,075	806.7	805
29	CT	MenACWY	1	63,091,662	60,119,537	815.3	802
30	CT	MenACWY	1	66,850,254	65,064,582	800	811
32	CT	MenACWY	1	52,133,887	48,229,727	872.7	830

Supplementary Table 3 – Sample size (breakdown by NAAT result, vaccine arm and time point). Vaccine type in NAAT-ve arm is irrelevant as neither the placebo or ChAdOx1 nCoV-19 vaccines would be expected to influence gene expression in a non-COVID illness but we have included vaccine type for completeness nevertheless: Stage 1

	D0			CT			CT+7		
	Luminex	3rd gen RNA-seq	SRNA/ next-gen RNA-seq	Luminex	3rd gen RNA-seq	SRNA /next-gen RNA-seq	Luminex	3rd genRNA-seq	SRNA /next-gen RNA-seq
**NAAT +ve total**	**12**	**3**	**3**	**16**	**16**	**16**	**13**	-	**12**
ChAdOx1 nCoV-19	5	2	2	7	7	7	5	-	5
MenACWY	7	1	1	9	9	9	8	-	7
**NAAT -ve total**	**11**	**7**	**8**	**17**	**13**	**13**	**7**	-	**7**
ChAdOx1 nCoV-19	4	3	3	8	5	5	4	-	3
MenACWY	7	4	4	9	8	8	3	-	4
**Total**	**23**	**10**	**10**	**33**	**29**	**29**	**20**	-	**19**

Supplementary Table 4 – Sample size (breakdown by NAAT result, vaccine arm and time point). Vaccine type in NAAT-ve arm is irrelevant as neither the placebo or ChAdOx1 nCoV-19 vaccines would be expected to influence gene expression in a non-COVID illness but we have included vaccine type for completeness nevertheless: Stage 2

	D0*		CT		CT+7	
	Luminex	SRNA/ next-gen RNA-seq	Luminex	SRNA /next-gen RNA-seq	Luminex	SRNA /next-gen RNA-seq
**NAAT +ve total**	**49**	**19**	**50**	**51**	**49**	**52**
ChAdOx1 nCoV-19	18	12	19	21	19	21
MenACWY	31	7	31	30	30	31

*D0 samples are collected from different participants and are not paired with CT and CT+7 samples for SRNA/mRNA seq. (For cytokine analysis, all time points are paired, except for one missing D0 and one missing CT+7).

Supplementary Table 5 – Summary demographics characteristics of study participants by study stage, NAAT result and vaccine arm: Stage 1

	All participants	ChAdOx1 nCoV-19		MenACWY		D0 controls*
		NAAT -ve	NAAT +ve	NAAT -ve	NAAT +ve	
N enrolled	34	9	7	9	9	11
Sex – Male (N, %)	17 (50%)	6 (66.7%)	4 (57.1%)	3 (33.3%)	4 (44.4%)	3 (27.3%)
Age – Years (Median, IQR)	35 (28, 45)	40.6 (28, 56.3)	32 (21, 45)	40 (32, 45)	34 (29, 40)	41.9 (21, 56.3)
BMI (Median, IQR)	26 (23.5, 29.1)	27 (23.9, 30.3)	28 (22.4, 29.1)	25 (23.5, 27.2)	25 (22.8, 37)	25 (23.7, 27.6)
Interval CT from prime – days (Median, IQR)	84 (55, 102)	69 (41, 76)	89 (71, 103)	69 (55, 81)	97 (93, 112)	-
Interval CT from boost – days (Median, IQR)	12 (6, 33)	8 (6, 37)	13 (3, 15)	5 (5, 5)	33 (14, 34)	-
Interval CT from symptom onset – days (Median, IQR)	3 (2, 4)	2 (1, 3)	2 (2, 3.5)	3.5 (1,75, 4)	4 (3, 6)	-
CT occurred before boost (N)	18	3	3	8	4	-
Illness severity**						
Mild (N)	27	8	5	5	9	-
Moderate A (N)	4	1	2	1	0	-

*D0 controls in stage 1 are taken from the subset of participants with NAAT results

**two-sided Chi-squared test comparing mild and moderate case proportions in the NAAT+ ChAdOx1 nCoV-19 and placebo groups yielded a p-value of 0.08.

Supplementary Table 6 - Summary demographics characteristics of study participants by study stage, NAAT result and vaccine arm: stage 2

	All participants	ChAdOx1 nCoV-19 NAAT+ve	MenACWY NAAT+ve	D0 controls*
N enrolled	71	21	31	19
Sex – Male (N, %)	19 (26.8%)	6 (28.6%)	6 (19.4%)	7 (36.8%)
Age – Years (Median, IQR)	37 (30, 49)	39 (32.1, 49)	37 (28, 46)	37 (29, 57.1)
BMI (Median, IQR)	27 (24.3, 33.1)	30 (24.3, 32.9)	28 (24.3, 35.5)	27 (24.5, 31)
Interval CT from prime – days (Median, IQR)	146 (130, 160)	141 (120, 160)	147 (136, 162)	-
Interval CT from boost – days (Median, IQR)	69 (48, 84)	74 (58, 84)	65 (45, 84)	-
Interval CT from symptom onset – days (Median, IQR)	2.5 (2, 4)	2 (2, 4)	3 (2, 4)	-
CT occurred before boost (N)	0	0	0	-
Illness severity**				
Mild (N)	40	15	25	-

Moderate A (N)	12	6	6	-

*D0 controls in stage 2 are independent controls from different participants of the vaccine trial.

**two-sided Chi-squared test comparing mild and moderate case proportions in the NAAT+ ChAdOx1 nCoV-19 and placebo groups yielded a p-value of 0.44.

Supplementary Table 7 – Contrasts used to create each volcano figure

Figure and plot title	Stage	Data type	contrast
3 b) NAAT+ve (placebo) Vs NAAT-ve	1	next-gen seq	CT_NAAT_positive_MenACWY - CT_NAAT_negative
3 d) NAAT+ve (placebo) Vs NAAT-ve	1	sRNA seq	CT_NAAT_positive_MenACWY - CT_NAAT_negative
4 d) D0 vs CT+7 placebo NAAT+ve	1	sRNA-seq	CT7_NAAT_positive_MenACWY – D0
5 a) D0 vs CT placebo NAAT+ve	1	next-gen seq	CT_NAAT_positive_MenACWY – D0
5 c) ChAdOx1 vs placebo NAAT+ve	1	next-gen seq	CT_NAAT_positive_MenACWY – CT_NAAT_positive_ChAdOx
5 e) D0 vs CT placebo NAAT+ve	1	sRNA-seq	CT_NAAT_positive_MenACWY – D0
5 f) ChAdOx1 vs placebo NAAT+ve	1	sRNA-seq	CT_NAAT_positive_MenACWY – CT_NAAT_positive_ChAdOx
5 h) ChAdOx1 vs placebo NAAT+ve	1	3rd gen RNA-seq	CT_NAAT_positive_MenACWY – CT_NAAT_positive_ChAdOx
6 b) Placebo D0 vs CT	2	next-gen seq	CT_NAAT_positive_MenACWY – D0
6 c) ChAdOx1 nCoV-19 D0 vs CT	2	next-gen seq	CT_NAAT_positive_ChAdOx – D0
6 f) CT ChAdOx1 nCoV-19 vs placebo NAAT+ve	2	next-gen seq	CT_NAAT_positive_MenACWY – CT_NAAT_positive_ChAdOx
7 a) Placebo D0 vs CT	2	sRNA-seq	CT_NAAT_positive_MenACWY – D0
7 b) ChAdOx1 nCoV-19 D0 vs CT	2	sRNA-seq	CT_NAAT_positive_ChAdOx – D0
S4 a) CT ChAdOx1 nCoV-19 vs placebo NAAT-ve	1	next-gen seq	CT_NAAT_negative_MenACWY – CT_NAAT_negative_ChAdOx
S4 b) CT ChAdOx1 nCoV-19 vs placebo NAAT-ve	1	3rd gen RNA-seq	CT_NAAT_negative_MenACWY – CT_NAAT_negative_ChAdOx
S4 c) CT ChAdOx1 nCoV-19 vs placebo NAAT-ve	1	sRNA-seq	CT_NAAT_negative_MenACWY – CT_NAAT_negative_ChAdOx
S24 a) D0 vs CT+7 placebo NAAT+ve	1	next-gen seq	CT7_NAAT_positive_ MenACWY – D0
S24 b) D0 vs CT+7 ChAdOx1 nCoV-19 NAAT+ve	1	next-gen seq	CT7_NAAT_positive_ChAdOx – D0
S24 c) D0 vs CT+7 NAAT-ve	1	next-gen seq	CT7_NAAT_negative – D0

S24 d) D0 vs CT+7 ChAdOx1 nCoV-19 NAAT+ve	1	sRNA-seq	CT7_NAAT_positive_ChAdOx – D0

Supplementary data

Supplementary Data 1 – Complete metadata per participant, information about the samples and time points collected for each of the omics datasets. Attached as Supplementary_Data_1_full_metadata_per_omics.xlsx

Supplementary Data 2 – DGE results, all contrasts, next-gen RNA sequencing, stage 1, attached as Supplementary_Data_2_RNA_seq_DGE_stage1.xlsx Differential expression analysis was performed using a two-sided moderate t-test.

Supplementary Data 3 – DGE results, all contrasts, next-gen RNA sequencing, stage 2, attached as Supplementary_Data_3_RNA_seq_DGE_stage2.xlsx Differential expression analysis was performed using a two-sided moderate t-test.

Supplementary Data 4 – DGE results, all contrasts, 3rd gen RNA sequencing, stage 1, attached as Supplementary_Data_4_ONT_RNA_seq_DGE_stage1.xlsx Differential expression analysis was performed using a two-sided moderate t-test.

Supplementary Data 5 – DGE results, all contrasts, small RNA sequencing, attached as Supplementary_Data_5_sRNA_seq_DGE_stage1.xlsx Differential expression analysis was performed using a two-sided moderate t-test.

Supplementary Data 6 – DGE results, all contrasts, small RNA sequencing, attached as Supplementary_Data_6_sRNA_seq_DGE_stage2.xlsx Differential expression analysis was performed using a two-sided moderate t-test.

Supplementary Data 7 – MIEAA2 GO BP results tables, small RNA sequencing, stage 1, attached as Supplementary_Data_7_MIEAA2_GO_BP_stage1.xlsx Significance testing obtained via MIEAA2 which uses a two-sided GSEA analysis approach.

Supplementary Data 8 – MIEAA2 GO BP results tables, small RNA sequencing, stage 2, attached as Supplementary_Data_8_MIEAA2_GO_BP_stage2.xlsx Significance testing obtained via MIEAA2 which uses a two-sided GSEA analysis approach.

Supplementary references

1 Griffiths-Jones, S., Grocock, R. J., van Dongen, S., Bateman, A. & Enright, A. J. miRBase: microRNA sequences, targets and gene nomenclature. *Nucleic Acids Res* **34**, D140-144 (2006). https://doi.org:10.1093/nar/gkj112

2 Yoshihama, M., Nakao, A. & Kenmochi, N. snOPY: a small nucleolar RNA orthological gene database. *BMC Res Notes* **6**, 426 (2013). https://doi.org:10.1186/1756-0500-6-426

3 O'Leary, N. A. *et al.* Reference sequence (RefSeq) database at NCBI: current status, taxonomic expansion, and functional annotation. *Nucleic Acids Res* **44**, D733-745 (2016). https://doi.org:10.1093/nar/gkv1189

4 Kern, F. *et al.* miEAA 2.0: integrating multi-species microRNA enrichment analysis and workflow management systems. *Nucleic Acids Res* **48**, W521-W528 (2020). https://doi.org:10.1093/nar/gkaa309

5 Chang, L., Zhou, G., Soufan, O. & Xia, J. miRNet 2.0: network-based visual analytics for miRNA functional analysis and systems biology. *Nucleic Acids Res* **48**, W244-W251 (2020). https://doi.org:10.1093/nar/gkaa467

6 Gennady, K. *et al.* Fast gene set enrichment analysis. *bioRxiv*, 060012 (2021). https://doi.org:10.1101/060012

7 Godard, P. & van Eyll, J. Pathway analysis from lists of microRNAs: common pitfalls and alternative strategy. *Nucleic Acids Res* **43**, 3490-3497 (2015). https://doi.org:10.1093/nar/gkv249

8 Chen, Y. *et al.* Conformational dynamics of dynamin-like MxA revealed by single-molecule FRET. *Nat Commun* **8**, 15744 (2017). https://doi.org:10.1038/ncomms15744

9 Jumper, J. *et al.* Highly accurate protein structure prediction with AlphaFold. *Nature* **596**, 583-589 (2021). https://doi.org:10.1038/s41586-021-03819-2
