# Supplementary material for: Multi-omics analysis reveals COVID-19 vaccine induced attenuation of inflammatory responses during breakthrough disease
Source: Nat Commun. 2024 Apr 22;15:3402. doi: 10.1038/s41467-024-47463-6 (PMC11035709; doi:10.1038/s41467-024-47463-6)
Supplement: Supplementary file 3 — Description of Additional Supplementary Files [file 41467_2024_47463_MOESM3_ESM.pdf]

Description of Additional Supplementary Files

File Name: Supplementary Data 1
Description: Complete metadata per participant, information about the samples and time points collected for each of the omics datasets.

File Name: Supplementary Data 2
Description: DGE results, all contrasts, next-gen RNA sequencing, stage 1. Differential expression analysis was performed using a two-sided moderate t-test.

File Name: Supplementary Data 3
Description: DGE results, all contrasts, next-gen RNA sequencing, stage 2. Differential expression analysis was performed using a two-sided moderate t-test.

File Name: Supplementary Data 4
Description: DGE results, all contrasts, next-gen RNA sequencing, stage 2. Differential expression analysis was performed using a two-sided moderate t-test.

File Name: Supplementary Data 5
Description: DGE results, all contrasts, small RNA sequencing. Differential expression analysis was performed using a two-sided moderate t-test.

File Name: Supplementary Data 6
Description: DGE results, all contrasts, small RNA sequencing. Differential expression analysis was performed using a two-sided moderate t-test.

File Name: Supplementary Data 7
Description: MIEAA2 GO BP results tables, small RNA sequencing, stage 1. Significance testing obtained via MIEAA2 which uses a two-sided GSEA analysis approach.

File Name: Supplementary Data 8
Description: MIEAA2 GO BP results tables, small RNA sequencing, stage 2. Significance testing obtained via MIEAA2 which uses a two-sided GSEA analysis approach.
