# Supplementary material for: Multi-omics analysis reveals COVID-19 vaccine induced attenuation of inflammatory responses during breakthrough disease
Source: Nat Commun. 2024 Apr 22;15:3402. doi: 10.1038/s41467-024-47463-6 (PMC11035709; doi:10.1038/s41467-024-47463-6)
Supplement: Supplementary file 12 — Reporting Summary [file 41467_2024_47463_MOESM12_ESM.pdf]

nature portfolio

Corresponding author(s): Daniel O'Connor

Last updated by author(s): Mar 26, 2024

Reporting Summary

Nature Portfolio wishes to improve the reproducibility of the work that we publish. This form provides structure for consistency and transparency in reporting. For further information on Nature Portfolio policies, see our Editorial Policies and the Editorial Policy Checklist.

Statistics

For all statistical analyses, confirm that the following items are present in the figure legend, table legend, main text, or Methods section.

n/a	Confirmed	
☐	☒	The exact sample size (*n*) for each experimental group/condition, given as a discrete number and unit of measurement
☐	☒	A statement on whether measurements were taken from distinct samples or whether the same sample was measured repeatedly
☐	☒	The statistical test(s) used AND whether they are one- or two-sided<br>*Only common tests should be described solely by name; describe more complex techniques in the Methods section.*
☐	☒	A description of all covariates tested
☐	☒	A description of any assumptions or corrections, such as tests of normality and adjustment for multiple comparisons
☐	☒	A full description of the statistical parameters including central tendency (e.g. means) or other basic estimates (e.g. regression coefficient) AND variation (e.g. standard deviation) or associated estimates of uncertainty (e.g. confidence intervals)
☐	☒	For null hypothesis testing, the test statistic (e.g. *F*, *t*, *r*) with confidence intervals, effect sizes, degrees of freedom and *P* value noted<br>*Give P values as exact values whenever suitable.*
☒	☐	For Bayesian analysis, information on the choice of priors and Markov chain Monte Carlo settings
☒	☐	For hierarchical and complex designs, identification of the appropriate level for tests and full reporting of outcomes
☐	☒	Estimates of effect sizes (e.g. Cohen's *d*, Pearson's *r*), indicating how they were calculated

Our web collection on statistics for biologists contains articles on many of the points above.

Software and code

Policy information about availability of computer code

Data collection	Randomisation lists were prepared by the study statisticians using block randomisation, stratified by study site and study group, and uploaded into a secure web platform used for the study electronic case report form (REDCap version 9.5.22)
Data analysis	STAR (version 2.7.3a), HTSeq (version 0.11.1), RNA2HLA (version1.1), bowtie (version 1.2.2), bowtie2(version 2.3.4.1), minimap (version 2.26), Salmon (version 1.10.1), CIBERSORTx , R programming language (packages edgeR v 3.32.1, limma v3.46.0, pcaExploer v2.16.0, ssRNA v1.3.2, fgsea v 1.27.1, ggplot2 v3.4.2, dplyr v1.1.3, biomaRt v2.46.3, tmod v0.50.13. Code is available at Github https://github/Chelysheva/COVID_multiomics_codes; https://github/dan-scholar/COVID_RNAseq_script

For manuscripts utilizing custom algorithms or software that are central to the research but not yet described in published literature, software must be made available to editors and reviewers. We strongly encourage code deposition in a community repository (e.g. GitHub). See the Nature Portfolio guidelines for submitting code & software for further information.

Data

Policy information about availability of data

All manuscripts must include a data availability statement. This statement should provide the following information, where applicable:

- Accession codes, unique identifiers, or web links for publicly available datasets
- A description of any restrictions on data availability
- For clinical datasets or third party data, please ensure that the statement adheres to our policy

The gene expression datasets are available on Gene Expression Omnibus (GSE228842; https://www.ncbi.nlm.nih.gov/geo/query/acc.cgi?acc=GSE228842).

Research involving human participants, their data, or biological material

Policy information about studies with human participants or human data. See also policy information about sex, gender (identity/presentation), and sexual orientation and race, ethnicity and racism.

Reporting on sex and gender	Biological sex information has been collected and is stored as part of the clinical trial data. 50% of stage 1 were male, and 27% of stage 2.
Reporting on race, ethnicity, or other socially relevant groupings	Race and ethnicity were collected as part of the clinical trial but are not report in this manuscript.
Population characteristics	Healthy volunteers aged 18-55 were recruited in the study of ChAdOx1 nCoV-19 vaccine - COV001 and COV002. All 105 participants included here had no history of laboratory confirmed SARS-CoV-2 infection prior to enrollment. Demographic characteristics of participants are available in supplementary tables.
Recruitment	Participants were recruited through local advertisements
Ethics oversight	South Central Berkshire Research Ethics Committee (20/SC/0145 and 20/SC/0179) and the UK regulatory agency (the Medicines and Healthcare products Regulatory Agency).

Note that full information on the approval of the study protocol must also be provided in the manuscript.

Field-specific reporting

Please select the one below that is the best fit for your research. If you are not sure, read the appropriate sections before making your selection.

- [x] Life sciences
- [ ] Behavioural & social sciences
- [ ] Ecological, evolutionary & environmental sciences

For a reference copy of the document with all sections, see nature.com/documents/nr-reporting-summary-flat.pdf

Life sciences study design

All studies must disclose on these points even when the disclosure is negative.

Sample size	The sample size was determined based on the availability of samples, especially those with breakthrough infection following vaccination. Baseline samples were not available for all participants, so unpaired analysis was conducted controlling for age, sex, body mass index and comorbidities. Power calculations were included.
Data exclusions	No data were excluded
Replication	The conducted two stages of analyses from the same overall sample set.
Randomization	The studies were observed-blinded randomised controlled trials (RCTs)
Blinding	The studies were observed-blinded randomised controlled trials (RCTs)

Reporting for specific materials, systems and methods

We require information from authors about some types of materials, experimental systems and methods used in many studies. Here, indicate whether each material, system or method listed is relevant to your study. If you are not sure if a list item applies to your research, read the appropriate section before selecting a response.

Materials & experimental systems

n/a	Involved in the study
☒	☐ Antibodies
☒	☐ Eukaryotic cell lines
☒	☐ Palaeontology and archaeology
☒	☐ Animals and other organisms
☐	☒ Clinical data
☒	☐ Dual use research of concern
☒	☐ Plants

Methods

n/a	Involved in the study
☒	☐ ChIP-seq
☒	☐ Flow cytometry
☒	☐ MRI-based neuroimaging

Clinical data

Policy information about clinical studies

All manuscripts should comply with the ICMJEguidelines for publication of clinical research and a completedCONSORT checklist must be included with all submissions.

Clinical trial registration	Registered on clinicalTrials.gov : NCT04324606 and NCT04400838
Study protocol	https://doi.org/10.1016/S0140-6736(20)32661-1, appendix 2
Data collection	All symptomatic COVID-19 cases occurred between June and December 2020. Recruitment for the trial started April 23rd 2020.
Outcomes	Participants presented for COVID-19 test visit (CT) during the study period as soon as possible after the onset of symptoms of COVID-19. At this visit, they were medically assessed, a COVID-19 test was performed and a blood sample was taken for analysis described in this study. They re-attended for the same procedure 7 days after their CT visit (CT+7)

Plants

Seed stocks	N/A
Novel plant genotypes	N/A
Authentication	N/A
